# Biomarker of food intake for assessing the consumption of dairy and egg products

**DOI:** 10.1186/s12263-018-0615-5

**Published:** 2018-09-29

**Authors:** Linda H Münger, Mar Garcia-Aloy, Rosa Vázquez-Fresno, Doreen Gille, Albert Remus R Rosana, Anna Passerini, María-Trinidad Soria-Florido, Grégory Pimentel, Tanvir Sajed, David S Wishart, Cristina Andres Lacueva, Guy Vergères, Giulia Praticò

**Affiliations:** 1 Agroscope, Bern, Switzerland; 2 Biomarkers and Nutrimetabolomic Laboratory, Department of Nutrition, Food Sciences and Gastronomy, XaRTA, INSA, Faculty of Pharmacy and Food Sciences, Campus Torribera, University of Barcelona, Barcelona, Spain; 3 CIBER de Fragilidad y Envejecimiento Saludable (CIBERFES), Instituto de Salud Carlos III, Barcelona, Spain; 4 Department of Biological Sciences, University of Alberta, Edmonton, AB T6G 2E9 Canada; 5 Epidemiology, Biostatistics and Prevention Institute, University of Zurich, Hirschengraben 84, 8001 Zurich, Switzerland; 6 University of Copenhagen, NEXS 30, Rolighedsvej, DK-1958 Frederiksberg C, Denmark; 7 Service of Endocrinology, Diabetology and Metabolism, Lausanne University Hospital, CHUV, Lausanne, Switzerland; 8 Department of Computing Science, University of Alberta, Edmonton, AB T6G 2E9 Canada

**Keywords:** Biomarkers of food intake, Food exposure markers, Dietary assessment, Dairy, Milk, Cheese, Yogurt, Whey, Casein, Eggs

## Abstract

Dairy and egg products constitute an important part of Western diets as they represent an excellent source of high-quality proteins, vitamins, minerals and fats. Dairy and egg products are highly diverse and their associations with a range of nutritional and health outcomes are therefore heterogeneous. Such associations are also often weak or debated due to the difficulty in establishing correct assessments of dietary intake. Therefore, in order to better characterize associations between the consumption of these foods and health outcomes, it is important to identify reliable biomarkers of their intake. Biomarkers of food intake (BFIs) provide an accurate measure of intake, which is independent of the memory and sincerity of the subjects as well as of their knowledge about the consumed foods. We have, therefore, conducted a systematic search of the scientific literature to evaluate the current status of potential BFIs for dairy products and BFIs for egg products commonly consumed in Europe. Strikingly, only a limited number of compounds have been reported as markers for the intake of these products and none of them have been sufficiently validated. A series of challenges hinders the identification and validation of BFI for dairy and egg products, in particular, the heterogeneous composition of these foods and the lack of specificity of the markers identified so far. Further studies are, therefore, necessary to validate these compounds and to discover new candidate BFIs. Untargeted metabolomic strategies may allow the identification of novel biomarkers, which, when taken separately or in combination, could be used to assess the intake of dairy and egg products.

**Electronic supplementary material:**

The online version of this article (10.1186/s12263-018-0615-5) contains supplementary material, which is available to authorized users.

## Background

The assessment of dietary intake with food frequency questionnaires, food diaries and 24 h recall methods is an important element of nutritional research as it allows to link the dietary behaviour of subjects enrolled in nutritional studies to their health status [1]. The application of these self-reporting tools is, however, limited by their subjective nature. Biomarkers of food intake (BFIs), on the other hand, belong to the class of dietary and health biomarkers and measure the intake of specific food groups, foods, or food components and can be used to estimate recent or average intake of these entities [2]. Combining the more classical assessment of dietary intake with robust, validated BFIs is therefore an obvious step towards improved assessments in nutritional studies [2–6].

The Joint Programming Initiative ‘A Healthy Diet for a Healthy Life’ (JPI-HDHL) Food Biomarker Alliance (FoodBAll) has developed a strategy [7, 8] and a methodology to define [2], identify [9] and validate [10] BFIs (http://foodmetabolome.org). In particular, FoodBAll aims at mapping the currently known or suggested BFIs for a range of foods across all major food groups, assessing their strengths and weaknesses and prioritizing the work needed to identify new biomarkers and to validate previous candidates, thereby providing much better tools for future studies in nutrition and health [7, 8]. Among these foods, foods of animal origin constitute a predominant part of Western diets [11]. Foods of animal origin can be primarily divided into (i) meat (mammals and birds) and meat products, (ii) fish, seafood and terrestrial animals other than mammals and birds, (iii) milk and dairy products, (iv) eggs and egg products and (v) animal fats and their primary derivatives [11, 12]. These foods are highly diverse in their characteristics (chemical composition, texture…), even for foods within each of these subcategories, rendering the analysis of their associations with nutritional and health outcomes difficult. A strategy taking into account this diversity is therefore necessary in order to identify BFIs that can ultimately support nutritional policies aimed at public health and disease prevention. This article focuses on BFIs for dairy products and BFIs for egg products. It is the first of two articles of the FoodBAll consortium in which potential BFIs for foods of animal origin commonly consumed in Europe are reviewed; the second article on fish and meat intake will be published separately.

Dairy products constitute a heterogeneous food group, which includes milk and milk proteins (casein and whey), fermented non-solid dairy products (sour cream, buttermilk, quark, kefir and yogurt), butter and cheese. The association of dairy products intake with disease outcomes is debated and often weak, except possibly for protection against osteoporosis [13]. However, the weak associations could very well be due to the heterogeneity of the composition of these products. Dairy products have been under criticism regarding their impact on health due to their high content in saturated fatty acids [14], salt (cheese) [15] and added sugar (e.g. yogurt) [16]. In particular, the relatively high intake of saturated fats that accompanies dairy intake has been speculated to result in increased cholesterol levels [17]. However, cheese intake seems to lower cholesterol compared to an equal amount of fat from butter indicating that other compounds in fermented dairy products may provide beneficial actions beyond those expected solely from the nutritional composition of these products [18]. On the other hand, a recent review of 52 clinical studies investigating the influence of dairy products on inflammatory markers concluded that dairy products have anti-inflammatory properties, particularly for persons suffering from metabolic diseases. This review provided evidence for an anti-inflammatory activity of both low- and high-fat dairy products, as well as for fermented dairy products [19]. Given the large variety of dairy products, there is a clear need to identify and validate specific BFIs that could discriminate between different dietary sources of these products.

Eggs and egg products represent another important group of foods of animal origin. Eggs, most commonly from hens, are a rich source of proteins, hence of amino acids, and several essential nutrients, particularly vitamin D, vitamin B12, vitamin A, selenium and choline. Eggs are also rich in cholesterol, but there is no strong association between egg intake and risk of CVD [20]. However, since eggs are hidden in many foods, associations between their consumption and health effects or diseases are difficult to establish and, therefore, remain uncertain. This fact points out the crucial need to find reliable biomarkers for egg intake.

In this manuscript, the most promising BFIs for dairy products and BFIs for egg products will be reviewed and evaluated for their suitability as biomarkers of intake, according to the validation scheme proposed by FoodBAll [10].

## Methods

### Selection of food groups

In order to obtain a good coverage of the foods of animal origin consumed in Europe, the three main classes of animal-derived products were taken into account: meat, including fish and seafood, dairy products and eggs. In the present review, we report the results from a systematic literature search for dairy products and eggs. Dairy products were subdivided into seven categories: milk, butter, cheese, yogurt, other fermented non-solid products, as well as whey and casein proteins. Therefore, including eggs, a total of eight food groups were reviewed for their respective biomarkers of intake.

### Primary literature search

The article selection, reviewing and reporting process made use of the methodology previously proposed from the FoodBAll consortium to carry out an extensive, systematic literature search and evaluation of BFIs [9]. In brief, original research papers and reviews were searched in at least two databases, including PubMed, Scopus and ISI Web of Knowledge, using combinations of the grouped search terms (biomarker* OR marker* OR metabolite* OR biokinetics OR biotransformation) AND (trial OR experiment OR study OR intervention) AND (human* OR men OR women OR patient* OR volunteer* OR participant*) AND (urine OR plasma OR serum OR blood OR excretion) AND (intake OR meal OR diet OR ingestion OR consumption OR eating OR drink* OR administration), as reported in Additional file 1: Table S1, together with specific keywords related to each animal-derived food group (Additional file 1: Table S2). The default search fields for each of the databases were [All Fields] for PubMed, [Article Title/ Abstract/ Keywords] for Scopus and [Topic] for ISI Web of Science, respectively. In the case of the search for “milk”, the field [Abstract/title] was used in the PubMed database to focus the search and reduce the number of irrelevant results. The literature search was conducted in November 2015. The search for dairy products, milk, cheese, milk protein and non-solid fermented product was updated in March 2017. In 2016, FoodBAll has initiated a series of well-defined standardized short-term interventions studies covering a range of fourteen foods [7], among these are milk and cheese [21, 22]. The data resulting from these studies has only been partially analysed and published and will therefore be evaluated at a later stage.

The search was limited to papers in the English language, while no restriction was applied for the publication date. The research papers identifying or using potential biomarkers of intake for the foods of animal origin were selected from the initial list of retrieved references by one or more skilled researchers in a process outlined in Additional file 2: Figure S1. The papers obtained from the search in different databases were merged and filtered for duplicates. Subsequently, papers were screened based on title and abstract. The selected papers were then retrieved and assessed for eligibility based on the contents of the entire manuscript. Additional papers were identified from reference lists in these papers and from reviews or book chapters identified through the search. The result was a list of compounds potentially relevant as biomarkers for the food group and corresponding references (Table 1 and Additional file 1: Tables S3–S9).

**Table 1:** List of reported putative biomarkers for the intake of dairy products

Dietary factor	Study design	*N*	Meth	Sample type	Discriminating metabolites/candidate biomarkers	Ref
Dairy products in general						
Dairy products	Randomized intervention study (increased dairy, reduced dairy or no change, 1 month)	180	GC-MS and GC-FID	Blood plasma	C15:0 in phospholipids	[32]
					C17:0 in phospholipids	
Dairy fat	Randomized controlled intervention study (regular fat dairy replaced by low-fat dairy, 12 weeks)	114 children	–	Blood serum	C15:0	[34]
Dairy products (cheddar cheese, butter, extra creamy whole milk)	Randomized, cross-over intervention (breakfast with dairy or soy, postprandial 4 h)	16	LC-MS (lipidomics)	Blood plasma	Phosphatidylcholine C15:0	[44]
					Phosphatidylcholine C17:0	
Dairy fat (from milk, sour milk, yogurt, cheese, cream and butter)	Case-control study/FFQ	111 cases + 107 controls	GC-FID	Blood serum (and adipose tissue)	C17:1 in non-esterified fatty acids, triacylgylcerols and cholesteryl esters	[28]
Dairy products (milk, yogurt, cheese, milk-based desserts, soymilk)	Observational data from 5 nested case-control studies	1336	LC-MS	Blood serum	Myristoyl-sphingomyelin SM(d18:1/14:0)	[59]
			GC-MS		Galactonate	
Cheese						
Cheese (yellow, hard cheese from cow’s milk, ‘Samsø’)	Randomized, crossover intervention study (6 weeks)	23	UPLC-QTOF/MS	Urine (24 h)	Isovalerylglutamic acid	[76]
					Isovalerylglycine	
					Tiglylglycine	
					Isobutyrylglycine	
Butter						
Butter	Nested case-control study/FFQ	502 (255 cases: incident colorectal cancer)	UPLC-MS	Blood serum	Methyl palmitate	[67]
			GC-MS			
Whey protein						
Whey isolate	Randomized, blinded, crossover meal study/acute study (8 h)	11	LC-QTOF/MS	Blood plasma	γ-glutamyl-leucine, γ-glutamyl-valine, γ-glutamyl-methionine, Tryptophan	[98]
Whey hydrolysate	Randomized, blinded, cross-over meal study/acute study (8 h)	11	BXY80615LC-QTOF/MS	Blood plasma	cyclic dipeptides (Pro-Thr, Phe-Val, Ile-Val, Leu-Val, Ala-Ile) and other AA metabolites (β-Asp-Leu, pGlu-Pro, pGlu-Leu, pGlu-Val),	[101]
Whey isolate				Blood plasma	γ-glutamyl-leucine	[101]

*N* number of subjects, *Meth* analytical method, *Ref* primary reference(s)

### Exclusion criteria

Exclusion criteria were specific for each food searched and are reported in Additional file 2: Figure S1. In general, papers were excluded if they investigated the effect on human physiology of the food considered, the presence or effect of toxicants, if they referred to unspecific markers or if they were based on in vitro or animal models.

### BFIs identification and classification

A second search step was used to evaluate the apparent specificity of the markers in the list. The compound databases HMDB [23] and FooDB (//foodb.ca) were used for the first evaluation of marker specificity. If the marker was not specific for a single food group, its relation to any foods outside the animal-derived food groups was documented. In the latter case, the compound was omitted from the list for the next search. The remaining list of potential biomarkers was used for a second literature search in the three bibliographic databases used for the primary search in order to identify other foods containing the potential biomarkers, or their precursors, as well as foods otherwise associated with these compounds. For the second web-based literature search, the “marker name” was used as a keyword, together with AND (biomarker* OR marker* OR metabolite* OR biokinetics OR biotransformation). Further filters, such as (urine OR plasma OR serum OR blood OR excretion) AND (intake OR meal OR diet OR ingestion OR consumption OR eating OR drink* OR administration) AND (human* OR men OR women OR patient* OR volunteer* OR participant* OR subject*), were added based on the results obtained. Again, markers related to foods of non-animal origin were deleted from the list. Potentially specific markers for each animal-derived food group or for several combined foods of animal origin are listed in Table 1, together with information about the study designs of the papers reporting their use. Due to the large number of studies, which suggested C15 and C17 as markers for dairy intake, only intervention studies were listed in Table 1 for these two metabolites. For all other markers identified, observational and interventional studies were considered in Table 1. The full list of studies for each food category is available in Additional file 1: Tables S3–S9. At the end of this selection process, the strength and weakness of each compound was evaluated and the most promising biomarkers were scored to assess their validity as BFIs according to the system reported below.

### Marker validation assessment

In order to further assess the validity of the biomarker candidates, a set of consensus evaluation criteria, from the FoodBAll consortium, was employed [10]. In particular, the usefulness of each marker was assessed by answering a set of simple questions (Additional file 1: Table S10), which reflect the analytical and biological criteria that the proposed biomarker should fulfil in order to be considered valid. Possible answers were Y (yes, the criterion is fulfilled for at least some use of the biomarker), N (no, the criterion has been investigated but it was not fulfilled), or U (uncertain, the criterion has not been investigated or data is not available). The potential markers were scored for chemical and biological plausibility (question 1), dose-response relationship, including saturation levels (question 2) and time-response after acute and repeated intake (question 3). The markers were further evaluated for their robustness in complex diets or real exposure situation (question 4) and reliability (question 5), that is the concordance with other measures of intake for the food or food group in question (such as other existing validated biomarkers or dietary instruments). The analytical aspects of each BFI were investigated through an evaluation of their chemical stability (question 6), their analytical performance (question 7) and reproducibility in different laboratories (question 8). This approach was applied to obtain an overview of the current level of validation of candidate BFIs and highlight which additional studies are needed to provide full validation of each candidate biomarker of animal-based foods [10].

## Results and discussion

### Biomarker of dairy products

After removal of duplicates, the original papers identifying or using potential biomarkers of intake for the different dairy products were selected from the list of retrieved references by two skilled researchers, following the process outlined in Additional file 2: Figure S1. In total, 1023, 730, 271, 196, 280, 329 and 153 publications were found using the above-defined search criteria for dairy products, milk, butter and cheese, yogurt, casein and whey, respectively. These papers were further screened for relevant markers. In the end, after the web-based literature search and the examination of the reference lists, only 23 papers contained relevant information about putative markers for dairy intake, 6 for milk, 3 for butter, 2 for cheese, 7 for yogurt, 3 for casein and 6 for whey intake. The search for non-solid fermented dairy products did not provide any relevant papers. Additional details regarding these studies are reported in Additional file 1: Tables S4–S9. The most promising candidates were selected and the validation criteria described by Dragsted et al. [10] were employed in each case (Table 2). In the following chapters, the characteristics of these candidate makers are presented.

**Table 2:** Validation scheme for candidate intake biomarkers of dairy products. The questions Q1-Q8 are reported in Additional file 1: Table S10. Possible answers are Y (yes, the criterion is fulfilled for at least some use of the biomarker), N (no, the criterion has been investigated but it was not fulfilled), or U (uncertain, the criterion has not been investigated or data is not available). The questions are based on the criteria of Dragsted et al. [10]

Food item	Metabolites	HMDB	Biofluid	Q1	Q2	Q3	Q4	Q5	Q6	Q7	Q8
Dairy fat/dairy products	C15:0	HMDB0000826	Blood plasma/serum	Y	U	N	Y	Y	U	Y	Y
	C17:0	HMDB0002259	Blood plasma/serum	Y	U	N	Y	Y	U	Y	Y
	C17:1	HMDB0060038	Blood serum	Y	U	N	Y	U	U	Y	N
Dairy products	Myristoyl-sphingomyelin SM(d18:1/14:0)	NA	Blood serum	Y	U	N	Y	U	U	U	N
	Galactonate	HMDB0000565		Y	U	N	Y	U	U	U	N
Butter	Methyl palmitate	HMDB0061859	Blood serum	Y	U	N	Y	U	U	U	N
Cheese	Isovalerylglutamic acid	HMDB0000726	Urine	Y	U	N	Y	U	U	U	N
	Isovalerylglycine	HMDB0000678	Urine	Y	U	N	Y	U	U	U	N
	Tiglylglycine	HMDB0000959	Urine	Y	U	N	Y	U	U	U	N
	Isobutyrylglycine	HMDB0000730	Urine	Y	U	N	Y	U	U	U	N
Whey protein hydrolysed	Cyclo (Phe-Val)	NA	Blood plasma	N	U	Y	U	U	U	U	U
	Cyclo (Ile-Val)/ Cyclo (Leu-Val)	NA	Blood plasma	N	U	Y	U	U	U	U	U
	Cyclo (Ala-Ile)	NA	Blood plasma	N	U	Y	U	U	U	U	U
	pGlu-Pro	NA	Blood plasma	N	U	Y	U	U	U	U	U
	pGlu-Leu	NA	Blood plasma	N	U	Y	U	U	U	U	U
	pGlu-Val	NA	Blood plasma	N	U	Y	U	U	U	U	U
Whey protein	γ -glutamyl-leucine	HMDB0011171	Blood plasma	U	U	Y	U	U	U	U	U
	γ-glutamyl-valine	HMDB0011172	Blood plasma	U	U	Y	U	U	U	U	U
	γ-glutamyl-methionine	HMDB0034367	Blood plasma	U	U	Y	U	U	U	U	U
Casein	Proline-proline	HMDB0011180	Blood plasma	U	U	Y	N	U	U	U	U
	Isoleucine-proline	HMDB0011174	Blood plasma	U	U	Y	N	U	U	U	U

#### Dairy products in general

We found that most studies focused on lipids when assessing biomarkers of dairy product intake. Based on observational data, the majority of these studies applied a targeted approach for fatty acid analysis using gas chromatography (GC) in order to evaluate the potential of two odd-chain fatty acids, pentadecanoic acid (C15:0) and heptadecanoic acid (C17:0), as biomarkers of dairy products or dairy fat [24–39]. The first study evaluating ruminant fatty acids as biomarkers for dairy fat intake based on dietary assessment showed that the content of C15:0 as well as C17:0 in adipose tissue might serve as a valid marker for long-term intake [40].

The high correlation between the levels of C15:0 in adipose tissue and dairy fat intake has been confirmed by subsequent studies [25–28]. Compared to adipose tissue, the concentration of C15:0 in blood lipids might be an equally good marker even though the correlation values were somewhat lower. A positive correlation between dairy fat intake and C15:0 in serum cholesterol esters has been observed in several studies [24, 25, 28]. Also, C15:0 in serum cholesterol esters has been associated with dairy product consumption in general [36]. An even greater number of observational studies has shown a positive association between C15:0 in phospholipids in serum or plasma and the intake of dairy fat [24, 25, 27, 35], dairy products [30, 35, 41], whole-fat dairy products [31, 42] and low-fat dairy products [42]. However, another study did not reveal a significant correlation between dairy fat intake and C15:0 in serum phospholipids or in free fatty acids but reported high correlation with C15:0 in cholesterol esters and serum triacylglycerols. Positive correlations in observational studies have also been demonstrated for C15:0 with total serum or plasma lipids and dairy fat intake [26, 29], as well as with dairy product intake [33, 38, 39]. Positive correlations were also found between C15:0 in dried blood spots and the intake of total, high-fat and low-fat dairy products, the correlation with high-fat dairy products being the strongest [37]. None of these studies used untargeted metabolomics except for the study by Nestel et al. [41]. In addition to several complex lipids identified as putative markers, C15:0 in phospholipids and in lysophosphosphatidylcholine showed a significant association with the number of dairy servings. The growing body of evidence from observational studies to use C15:0 as a biomarker of dairy fat or dairy products intake has been complemented by the outcomes of recent intervention studies. In another study, increasing dairy intake resulted in a small increase in plasma C15:0 levels whereas decreasing dairy intake was not associated with a change in the level of C15:0. In contrast, the replacement of full-fat dairy foods by reduced-fat or low-fat products was associated with decreased levels of serum C15:0, indicating that blood level of C15:0 is sensitive to changes in the absolute intake of dairy fat.

The effect of dairy fat on blood lipids upon short-term intake was assessed in 124 healthy volunteers consuming a daily three servings of dairy products or control products [39]. After 4 weeks, the fatty acid profiles, as analysed by targeted GC-FID, revealed that the plasma levels of C15:0 were significantly higher when compared to the intake of non-dairy control products. Postprandial effects were investigated by comparing plasma phospholipids profiles using a LC-MS lipidomics approach, which revealed that phosphatidylcholine containing C15:0 showed a significantly larger plasma increase after the dairy-based meal compared to the soy meal.

Several observational studies investigating the association between dairy intake and C15:0 in blood also included C17:0 analyses. Some of these studies showed that C17:0 in cholesterol esters [25], C17:0 in phospholipids [25, 27, 35] and C17:0 in lysophosphatidylcholine [41] were associated with dairy fat intake. Controversially, the study by Smedman et al. [24] did not reveal a correlation of serum C17:0 phospholipids to milk fat intake, while Brevik et al. [26] even reported an inverse association between serum C17:0 and dairy fat intake. The correlation factors between plasma C17:0 and dairy fat and dairy products intake were consistently lower when compared to C15:0 or even to *trans*16:1n-7 [29]. Albani et al. [37] reported a positive correlation of C17:0 in whole blood samples with the intake of total, high-fat and low-fat dairy products. The correlation was however lower in comparison to C15:0. Interestingly, a cross-sectional study including 2380 participants and using targeted metabolomics proposed serum lysophosphatidylcholine C17:0 as a specific marker of dietary pattern consisting of high intake of high-fat dairy and butter combined with a low margarine intake [43]. The intervention studies devoted to C15:0 in the former paragraphs also showed an effect of dairy products on serum C17:0 phopshatidylcholine levels during a 4 h postprandial period [44], on plasma levels of C17:0 after 4 weeks of daily intake of three dairy servings [39] as well as after an increased dairy intake [32]. However, the levels of serum C17:0 were not affected by replacing full-fat dairy with low-fat dairy products [34]. Overall, C17:0 seems to be a less sensitive marker of dairy intake in comparison to C15:0.

Finally, a cross-sectional study associating phospholipid fatty acids with food intake reported that the sum of plasma C15:0 and C17:0 phospholipids was not associated with dairy products intake at the population level but an association was found at the individual level. This finding suggests that the combination of several nutrients with an individualization of data analysis may represent promising strategies for the identification of specific markers of food intake.

Nevertheless, due to inconsistent outcomes, it remains unclear if the use of odd-chain fatty acids as biomarkers for dairy intake is limited to dairy fat intake only or to the intake of dairy products in general. In addition, a randomized, double-blind crossover study in 16 healthy individuals showed that supplementation with inulin or propionate for 7 days significantly increased pentadecanoic acid and heptadecanoic acid [45], indicating that the endogenous production of these molecules may be a confounder for their use as BFIs. Moreover, concern has been expressed regarding the use of odd-chain fatty acids as markers of dairy products as these molecules also occur in certain types of fish and in meat [46, 47]. Indeed, Saadatian-Elhani et al. [48] reported a strong positive correlation between the total intake of fish and plasma C17:0 whereas there was no correlation to dairy product intake at the population level. On the other hand, a metabolic analysis by Floegel et al. [43] found that the intake of dairy products, but not the intake of fish, was associated with lysophosphatidylcholine C17:0. Also, in other studies investigating both fish and dairy products, no correlation between odd-chain fatty acids and fish intake was reported [35, 36]. In conclusion, in populations consuming high amounts of fish, the intake of C15:0 and C17:0 through fish should not be neglected in studies targeting markers for the intake of dairy products in order to avoid misleading conclusions [46].

Aside from C15:0 and C17:0, a range of other lipids or fatty acids were also proposed as specific biomarkers of dairy intake. Heptadecaenoic acid (C17:1) has been found to strongly correlate with the total intake of dairy products in one study [28] but was not investigated in any further studies. Apart from odd-chain fatty acids, other ruminant fatty acids correlate with dairy intake. *Trans*-palmitoleic acid (*trans*-16:1n-7) in plasma total lipids correlates well with the intake of dairy fat [29] whereas plasma *trans*-16:1n-7 in phospholipids is associated with full-fat dairy intake [41, 42], but not with low-fat dairy intake [42]. Abdullah et al. [39] reported no effect on *trans*-16:1n-7 levels after 4 weeks’ intake of low-fat products. Erythrocyte *trans*-18:1 isomers were significantly correlated with total dairy consumption [49, 50]. However, some of these *trans*-fatty acids were also found in other foods and may thus be excluded from the list of potential markers of dairy intake. Metabolites present in other foods include *trans*-16:1n-7, which occurs in other ruminant-based foods, in particular, meat, at significant levels [51]. This compound can even be endogenously synthesized by humans from dietary *trans*-18:1 [52]. *Trans*-18:1 isomers may also derive from multiple foods produced with partially hydrogenated vegetable oils [51].

Phytanic acid is another fatty acid found in ruminant products. In a cross-sectional study, the strongest determinant of plasma phytanic acid concentration appeared to be dairy fat intake but a positive correlation was also found for ruminant meat [53]. Due to the lack of intervention studies and the use of metabolomics methods, it remains questionable if certain *trans*-fatty acids or phytanic acid are specific enough to be used as dairy markers or if they may rather serve as ruminant food markers. A correlation to the intake of dairy products or dairy fat has also been shown for myristic acid (C14:0) [25, 27, 32, 35, 36, 42]. However, this fatty acid occurs in most of the animal and vegetable fats and, thus, remains unspecific. Other unspecific fatty acids, which were shown to correlate to the intake of dairy products are C18:0 and C18:1n-9 [36], as well as C18:3n-6, C22:1n-9 and C22:5n-3 [39]. Other metabolites associated with dairy consumption have been identified but were not yet extensively studied. Nestel et al. [41] demonstrated that phospholipid C18:1n-7 (vaccenic acid) correlated with the number of full-fat dairy servings while two molecular forms of lyso-platelet-activating factor (C20:0 and C22:1) directly correlated with the number of dairy servings. Phosphatidylcholines C29:0, C31:0, C35:1, C28:0, C30:0, C32:0 and C32:1 showed a significantly larger increase in the postprandial period after the dairy meal compared to the soy meal possibly being indicative for dairy consumption [44]. More studies validating and confirming these metabolites for dairy intake are, however, required.

Another study showed that plasma trimethylamine-N-oxide (TMAO), a metabolite deriving from choline, was positively associated with milk and dairy food consumption [54]. Controversially, a 2-week intake of diets high in cheese and high in milk even decreased urinary TMAO levels compared to the control diet as reported by Zheng [55]. These authors suggest that cheese and milk modify the gut microbiota thus decreasing TMAO formation. Also, other experiments demonstrated that plasma TMAO was associated with several foods including egg intake [56], whereas urinary TMAO was associated with salmon intake [57]. Of note, TMAO is particularly interesting with regards to current discussions on its potential use as a prognostic factor for heart failure [58].

Based on an untargeted multi-platform—omics approach, a most recent study observed a positive correlation between serum myristoyl-sphingomyelin SM(d18:1/14:0), as well as galactonate and the intake of dairy products in fasting samples from nested case-control studies [59]. These metabolites represent two novel candidate markers and both deserve more investigation. Sphingomyelin is a component of the milk-fat globule and makes up about one third of total milk phospholipids [60], showing high potential to be specific to the intake of fat-containing dairy products. SM(d18:1/14:0) has been shown to be postprandially increased after a dairy meal and was decreased after a soy meal [61]. However, other animal-derived foods (meat, fish and egg) are also sources of sphingolipids [62], so that levels of SM(d18:1/14:0) influenced by other foods cannot be ruled at this point. On the other hand, galactonate derives from galactose metabolism and may possibly only be formed after ingestion of dairy products still containing high levels of galactose or lactose, respectively, thus non-fermented products.

#### Milk

Milk is a highly complex food providing essential macronutrients, vitamins, trace elements and minerals as well as many bioactive components. These may act synergistically to modulate metabolic and immunomodulatory processes, which are important for the maintenance of human health [61]. Pasteurized full-fat milk is mainly composed of water, followed by lactose, fat and proteins [63]. However, the exact composition of milk depends on several factors including feeding practices, breed, age and health status of the animals [61]. Technological treatments and storage conditions introduce further variations in macro- and micronutrient concentrations. As milk in its raw or heat-processed form is the starting material for a broad range of products, the challenge in identifying a marker compound specific for its intake is that it should not be present after intake of any other dairy product. Even though the health benefits of milk have been well documented, investigations on metabolites in human biofluids linked to milk intake are still scarce as most studies focus on dairy products in general.

First, three intervention studies that have used milk as a control or a test product are considered. The studies were conducted in children (high milk or high meat diet during 7 days) [64], in patients suffering from irritable bowel syndrome (probiotic yogurt or non-fermented acidified milk during 8 weeks) [65, 66] or in healthy adults (diet high in milk, high in cheese, or a control diet for 14 days) [55]. The potential biomarkers of milk intake identified included serum SFCA [64], L-lactate, 3-hydroxybutyrate, glutamine, proline, creatine/creatinine, aspartic acid [65, 66], urinary citrate and faecal glycerol [55].

Then, two observational studies treated milk as a separate variable by not including it in the general category of dairy products. Using food frequency questionnaires from 502 men and women aged 55–74 years, the first study could associate serum homostachydrine (also known as pipecolic acid betaine) with milk intake [67]. The second study could associate the intake of full-fat cow milk in toddlers with serum pentadecanoic acid C15:0, palmitic acid C16:0 and conjugated linoleic acid (CLA) [68].

However, the potential of using the above-mentioned metabolites as markers of milk intake remains elusive because of their lack of specificity. Several are involved in endogenous, biochemical reactions in humans, such as glycerol, which is released during the breakdown of triacylglycerols and phospholipids, citrate, which is formed in the tricarboxylic acid cycle, or free amino acids. Others can be influenced by dietary components other than milk. Blood SCFA can be influenced by colonic fermentation of fibres from cereals [69, 70], lactate and 3-hydroxybutyrate have been observed after the ingestion of wheat bran [71] and decaffeinated green tea extract [72], while C16:0 is one of the most common saturated fatty acids in animal and plant-based foods. As previously described, C15:0 may possibly serve as a biomarker of dairy fat or dairy products but it is not specific for milk. Homostachydrine is a plant metabolite occurring in, for example, alfalfa and citrus fruits [73]. The concentration of this specific compound in humans having ingested dairy products may thus depend on its content in milk, which in turn may depend on the feed supplied to the cattle. CLA, which can be formed by ruminal bacteria from unsaturated fatty acids, is mainly found in ruminant foods such as meat and milk, milk fat being dominated by *cis*-9, *trans*-11 CLA isomers. It might thus be a marker for ruminant foods, which would need validation against other CLA-containing foods of non-ruminant sources, for instance, egg. Nonetheless, the synthesis of CLA is not restricted to ruminal microbial activity as it can also be synthesized in humans through desaturation of vaccenic acid [74], which is a fatty acid also present in milk.

Finally, the studies mentioned here differ in the use of semi-skimmed [55, 65, 66] or skimmed milk [64], and information on heat treatment has only been supplied by the studies from Pedersen et al. [65, 66] who administered high-pasteurized milk. Such technological treatments have to be taken into account when evaluating the validity of potential biomarkers of intake.

#### Butter

Butter is a traditional dairy food being consumed worldwide, either pure in the form of a spread or processed in pastes or bakery products, as well as for refinement of foods and dishes. Besides water, proteins, lactose, lactic acid, vitamins and minerals, butter consists of approx. 80% of fat (mainly triacylglycerols), which is characterized by a broad fatty acid distribution ranging from short-, middle- to long-chain saturated and unsaturated molecules [63, 75].

Hjerpsted et al. [76] reported a 6-week intervention study applying a randomized crossover design investigating the effects of butter (control) and cheese (intervention) on urinary metabolites. Urine samples were analysed using an untargeted LC-MS approach. By applying univariate statistics and comparing the urine metabolome after daily cheese and butter consumption, seven metabolites were related to the intake of butter. However, these metabolites were only characterized by their mass-to-charge ratio in either positive or negative mode and their identification remains unknown.

Two recent observational studies investigated metabolites attributed to the intake of butter based on food frequency questionnaires [43, 67]. A cross-sectional study assessing associations between serum metabolites and habitual diet analysed by a targeted approach revealed that a diet with high butter but low margarine intake was associated with the presence in serum of acylcarnitines, acyl-alkyl-phosphatidylcholines, lysophosphatidylcholines and hydroxy-sphingomyelins [43]. Monounsaturated diacyl-phosphatidylcholines (C36:1, C28:1 and C34:1) were associated with high butter and sauce intake, whereas acyl-alkyl-phosphatidylcholines (C34:0, C36:1, C34:1 and C30:0) were associated with high intake of butter as well as red meat and high-fat dairy products. Lysophosphatidylcholines were associated with butter but also high-fat dairy and non-dairy sweet spreads, while sphingomyelin was associated with butter but also garlic and coffee intake. Only saturated acylcarnitines C9:0, C16:0 and C18:0 may have the potential to specifically serve as biomarkers for a diet with high butter intake. High levels of these acylcarnitines can be explained by the fatty acids provided through the intake of butter, whose composition is dominated by C16:0 and C18:0. However, the concentration of total acylcarnitines in blood is influenced by the dynamics of the human metabolome: they strongly increase during fasting period or exercise (catabolic state) due to elevated beta-oxidation of fatty acids in order to provide energy [77]. Moreover, acylcarnitine C16:0 is also elevated in the metabolic profile of meat consumers [78].

In a nested case-control study with cases suffering from colorectal cancer, correlations between metabolites and diet were assessed [67]. Three metabolites positively correlated with butter intake, namely methyl palmitate, pentadecanoic acid (C15:0) and 10-undecenoate. However, no differences in the association between the three molecules and dietary intake were observed by disease status, underlying the robustness of these putative markers as markers of dietary intake rather than markers of the health status. Methyl palmitate is a natural fatty acid methyl ester, which has been reported to occur in raw milk [79] and has not yet been found to be influenced by other dietary factors. On the other hand, although C15:0 was associated with butter intake, this molecule should rather be discussed as a marker for the intake of high-fat dairy products in general as discussed in the previous sections. Finally, 10-undecenoate is a monounsaturated medium-chain fatty acid with antifungal activity obtained by pyrolysis from castor oil [80], which had not been previously associated with dairy products; thus, its link to butter intake remains unclear.

#### Cheese

Cheese is mostly composed of proteins, fat and water. Fermentation with different species of lactic acid bacteria but also technological processes have led to a large variety of cheese types. Compounds derived from the fermentation process possess the greatest potential as biomarkers of cheese intake. However, the occurrence and concentration of such metabolites vary depending on many factors such as selection of the starter culture and ripening stage. The major challenge in the identification of biomarkers of cheese intake is the identification of a general marker representing the huge variety of cheese types.

Only few studies have been published on the identification of biomarkers of cheese intake. In a 6-week intervention study, based on untargeted LC-MS analyses, a high number of discriminating metabolites for cheese intake were identified when compared to butter intake [76]. Among these were urinary indoxyl sulfate, xanthurenic acid, tyramine sulfate, 4-hydroxyphenylacetic acid, isovalerylglutamic acid, isovalerylglycine, tiglylglycine and isobutyrylglycine. Indoxyl sulfate is synthesized from indole in the liver, a metabolite from tryptophan formed by the gut microbiota. Xanthurenic acid, formed via the kynurenine pathway, is another metabolite derived from tryptophan catabolism. The release of tryptophan during the cheese making process is attributed to the proteolytic activity of bacterial enzymes. However, increased levels of urine and plasma indoxyl sulfate have also been observed after the ingestion of a high-protein diet based on non-fermented milk proteins [81]. Urinary xanthurenic acid has been found to be a discriminating marker after cocoa powder with milk consumption [82]. Tyramine sulfate and 4-hydroxyphenylacetic acid derive from tyramine, an undesirable biogenic amine, which is formed by the microbial decarboxylation of free tyrosine during cheese ripening. Due to the detrimental effect of biogenic amines on health, their levels are kept as low as possible by optimizing technological processes. Thus, the potential use of tyramine-derived metabolites as a general biomarker of cheese intake is inappropriate. Furthermore, biogenic amines are not only restricted to cheese but also occur in fish and other fermented foods [83]. Metabolites derived from biogenic amines might thus rather represent biomarkers of fermented foods or as markers for quality issues during production. The formation of 4-hydroxyphenylacetate, another marker identified by Hjerpsted et al. [76], is also linked to gut microbiota metabolism of phenolic compounds; its levels in urine are changed after a vegetarian diet [84], wine intake [85] and the consumption of green tea [86]. Isovalerylglycine and tiglylglycine are metabolites derived from the catabolism of leucine and isoleucine, respectively. Isobutyrylglycine might derive from isobutyric acid, an aroma compound in cheese, or might be formed through valine catabolism [76]. So far, the occurrence of acylglycines in urine has not yet been linked to the consumption of other foods and thus these molecules remain interesting as potential biomarkers for cheese intake.

In a second intervention study, 15 volunteers consumed a diet high in semi-skimmed milk or cheese and a control diet for 14 days [55]. Based on NMR metabolomics analyses, cheese intake could be discriminated from milk intake and the control diet by significantly increased levels of urinary proline betaine and tyrosine. Tyrosine concentration in cheese increases due to the proteolytic activity of bacteria during processing and ripening. The authors thus suggest using tyrosine and proline betaine as exposure markers for cheese intake. However, the urinary concentration of proline betaine is significantly influenced by the consumption of citrus fruits and is considered a valid biomarker for citrus fruit intake [87–90]. On the other hand, tyrosine is a non-essential amino acid and elevated levels of urinary tyrosine have also been observed after cocoa consumption [91]. Therefore, neither proline betaine nor tyrosine is sufficiently specific as markers of cheese intake. In the same study, the consumption of both cheese and milk increased short-chain fatty acids (SCFA) when compared to the control diet in faecal samples. Furthermore, cheese intake was discriminated from milk intake by an increase in faecal butyrate. These observations can be explained by the direct intake of SFCAs, as both products contained SFCAs in different concentrations, but also by a more complex effect involving the metabolic activity of the gut microbiota on ingested nutrients [55]. For instance, butyrate levels in faeces are modified by the intake of dietary fibres [70, 92]. Therefore, SCFAs cannot be considered as specific biomarkers for cheese intake.

In order to obtain validated biomarkers for cheese intake, more studies with a variety of cheese types are required and potential biomarkers of cheese intake need to be validated against markers of intake of other fermented foods rich in protein such as yogurt.

#### Fermented non-solid dairy products

The use of differently processed milk as starting material (e.g. skimmed milk for quark, cream for sour cream, etc.) in addition to differences in the fermentation process (e.g. yeast fermentation in case of kefir) might lead to the formation of biomarkers enabling the discrimination of fermented non-solid dairy products from yogurt and cheese. Primary search on biomarkers of the intake of fermented non-solid products such as quark, sour cream, kefir and buttermilk, however, did not identify relevant research publications. Evidently, there is a lack of studies investigating intake biomarkers from fermented dairy products other than yogurt and cheese.

#### Whey protein and casein

Whey protein (WP) is a mixture of globular proteins isolated from whey, the liquid material obtained as a by-product of cheese production. WP is typically released in the market as four major forms, which differ in the production process and nutritional content, namely concentrate (WC), isolate (WI), hydrolysate (WH) and native whey: WC has a lower level of fat and cholesterol, but higher level in carbohydrates and lactose, as well as bioactive compounds; WI is processed to remove the fat and lactose, but is lower in bioactive compounds; WH is partially hydrolysed to facilitate the digestion and uptake of amino acids derived from its proteins; native whey is obtained from skim milk as a by-product of cheese production [93–96]. Casein (CAS) refers to a group of phosphoprotein (αS1, αS2, β, κ) commonly found in mammalian milk. It represents the major component of cheese, but it is also used as a food additive and protein supplement. Compared to WP, casein is richer in arginine, methionine, phenylalanine, proline and tyrosine [97, 98]. As for the other dairy products, different preparation methods, nutritional content and metabolic processing by the human organism render the discovery of candidate BFIs for such food products difficult.

When the intake of test drinks containing WP, WI, WH and CAS was compared with those of other carbohydrate-based drink or other protein sources, a general increase in plasma amino acids, urea and ammonia was observed, which reflected the different amino acid contents of the test proteins and the controls [99–101]. Furthermore, differences in postprandial peripheral amino acids profile reflected the absorption and the first pass metabolism [98, 102]. These results and the fact that amino acids are unspecific and present in large amount in many foods indicate that they cannot be used as a biomarker for the assessment of dairy protein intake.

A finding of the study of Stanstrup et al. [101] was the detection of methionine oxidation products, methionine sulfoxide (MetSO) and its metabolites, N-phenylacetyl-methionine (PAM) and N-phenylacetyl-methionine sulfoxide (PAMSO), in plasma samples after WH intake. These results were explained by the elevated presence of MetSO already in the powder material, as a result of the manufacturing process that must have caused the oxidation of methionine to the sulfoxide, while PAM and PAMSO could be generated endogenously. Interestingly, in a subsequent study [98], the same compounds were elevated after the CAS meal but not after the WI meal. PAM and PAMSO were elevated in urine, while PAM was also high in plasma. The authors ascribed again such result to the meal composition and the manufacturing process, which produces MetSO. As MetSO has previously been quantified in milk protein products showing a highly variable range of concentrations [103], such compounds seem not to be reliable markers to monitor dairy protein intake.

Another compound found in the two studies of Stanstrup et al. [98, 101] was β-Asp-Leu, which was elevated in plasma after the WH meal [101] as well as in urine after CAS intake [98]. Of note, this compound should not be present in dietary sources and should also not enter the bloodstream, as β-aspartate has been found to potently increase the immunogenicity of proteins in pharmaceuticals [104].

More promising compounds were found by Stanstrup et al. [98, 101] following the intake of the WP or CAS meal. The identified metabolites were several cyclic dipeptides and other amino acid metabolites, such as linear dipeptides or γ-glutamyl conjugates. The same cyclic dipeptides were also identified in the powdered product and could be classified into two groups, 2,5-diketopiperazines (DKPs) and pyroglutamyls. DKPs are side products of terminal peptide cleavage [105] created when the free α-amine in one amino acid and the α-carboxylic acid in the adjacent amino acid react to form a diketopiperazine ring. In particular, cyclo(Pro-Thr), cyclo(Phe-Val), cyclo(Ala-Ile) and a compound which could be either cyclo(Leu-Val) or cyclo(Ile-Val) showed a significant increase in plasma after WH intake [101]. The pyroglutamyls identified were pGlu-Pro, pGlu-Leu and pGlu-Val. Such compounds are formed from glutamic acid by reaction of the side-chain carboxylic acid with the α-amine in the same glutamic acid moiety to create a 5-oxoproline ring structure and were higher in plasma, as well, after the WH meal [101]. Interestingly, none of these compounds have been discriminating for WI or CAS in the second study [98] that suggests a specific correlation with WH. Although these cyclic dipeptides have already been found in several foods, such as cocoa [106], roasted coffee [107], beef [108] and wheat gluten [109], no other studies have investigated the presence of such compounds in human body fluids.

In the same study, two markers for caseinoglycomacropeptide (CGMP) intake were Pro-Pro and Ile-Pro [101], which could derive from Ile-Pro-Pro, a tripeptide found in CAS [110]. However, these dipeptides were not confirmed for CAS in the second study [98], suggesting that they may not be as robust as BFIs.

The levels of the intermediate metabolite γ-glutamyl-leucine were also different between meals in both Stanstrup’s studies [98, 101]. γ-glutamyl-leucine was higher after WI intake, but not after the casein-containing meals [98, 101]. The authors suggested that this discrepancy could be directly related to the elevated levels of the parent amino acids in the meal [98]. Although γ-glutamyl-leucine may reflect the proteolytic breakdown products of larger proteins, it has also been found in vegetables of the onion family (//foodb.ca).

#### Yogurt

Only few studies relate the consumption of yogurt to compounds detected in biosamples. Among these, a randomized crossover study comparing the effects of fresh versus heated yogurt in males with and without lactose malabsorption, reported an increase in plasma propionate in men with lactose malabsorption after fresh yogurt intake during 2 weeks, as well as a higher AUC of plasma butyrate over 3 h after a load of fresh yogurt in men without lactose malabsorption. However, no significant change in plasma acetate was observed.

A parallel study evaluating the effect of a probiotic yogurt on the serum metabolome of 61 patients with inflammatory bowel syndrome could not observe any modification after an 8-week intake compared to a non-fermented acidified milk [65]. In particular, an increase in serum lactate and 3-hydroxybutyrate was observed in both control and test groups, which raised the question of the neutrality of the delivering vector (milk in this case).

In an intervention crossover study, Fabian and colleagues [111] identified a significant increment of plasma thiamine and riboflavin in young healthy women after a 4-week intervention with yogurt consumption. The authors suggested that yogurt may contribute to the intake of both vitamins, due to the capacity of some specific strains of bacteria to synthesize B vitamins in fermented milk products [111].

Mohammad and co-workers [112] carried out a controlled randomized clinical trial using yogurt with and without *Lactobacillus acidophilus* fortification in children for 6 weeks. They demonstrated that the consumption on a daily basis of the supplemented yogurt significantly increased the levels of plasma vitamin B12. Similarly, the research held by Samuel et al. [113] pointed out that yogurt intake was significantly associated with plasma vitamin B12 status on a group of pregnant women with impaired vitamin B12 status. The vitamin B12 status depends on dietary intake, gut microbiota and the efficiency of its enterohepatic circulation [112]. Therefore, the positive association between yogurt intake and plasma levels of vitamin B12 could be related to the probiotic function of this food, since supplementation with probiotics has shown to improve the vitamin B12 status [112, 114]. In this field, it is important to point out that some intestinal bacterial strains have the capability to synthesize vitamin B12 in the small bowel [115]. Therefore, some bacteria from the probiotics could have the capability to colonize in the small bowel and produce vitamin B12 [114]. On the other hand, some Lactobacilli strains can synthetize vitamin B12 during the fermentation process, providing additional amounts of this vitamin when these fermented food are consuming [113].

As an alternative to circulating or faecal metabolites, changes in the host microbiota could also be proposed as a biomarker of yogurt intake. Such changes could happen either directly, with the survival of *S. thermophilus* and *L. bulgaricus* in the host’s GIT after yogurt intake, or indirectly, through modification of the existing microbiota. *S. thermophilus* and *L. bulgaricus* have non-human origins and low abilities to survive in gastric juice and adhere to intestinal epithelial cells [116, 117]. Therefore, their presence in the host’s microbiota highly relies on environmental exposure, in particular, the diet. The survival and growth of *S. thermophilus* and *L. bulgaricus* into the GIT has been confirmed by Burton et al. [118], Mater et al. [119] and Elli et al. [117] who recovered both strains from the faeces of healthy volunteers after daily yogurt intake during 14, 12 and 7 days, respectively. However, *S. thermophilus* and *L. bulgaricus* are widely used by the fermented food industry, compromising their specificity. Semi-hard cheeses such as Emmental and Gruyere or hard Italian varieties require the use of thermophilic starter cultures including *S. thermophilus* [120] and *L. bulgaricus* is used for mozzarella, provolone, Parmesan, Romano and Swiss cheeses productions. Therefore, the presence of these bacterial species in human’ faeces does not necessarily indicate yogurt intake.

Other groups of studies showed significantly increased levels of non-starter bacterial strain after the consumption of yogurt. Bartram and colleagues [121] reported an increased excretion of bifidobacteria after consumption during 3 weeks of yogurt enriched with *Bifidobacterium longum* and lactulose as well as after consumption by 12 healthy volunteers of a conventional yogurt in a double-blind randomized crossover study. Similarly, Hussein et al. [122] showed an increase in bifidobacterial and lactobacilli populations after daily consumption by 28 male volunteers during 3 weeks of a regular yogurt in a parallel study. Finally, Burton et al. [118] reported an increased in faecal Bifidobacteria after consumption during 2 weeks of a non-fermented milk acidified with D-(+)-glucono-δ-lactone but not after the consumption of yoghurt. In this field, it is, however, important to keep in mind that there are different methods and no standard protocols for assessing bacterial proportions in faecal samples [123].

Although the compounds mentioned in this section have been linked to yogurt consumption, none of them qualify as a biomarker of yogurt intake; these metabolites are widely distributed in several food sources, compromising their specificity with respect to yogurt intake. These compounds can more likely be used as biomarkers of nutrient intake, nutritional status or microbiota integrity than as biomarkers of specific food consumption, as recently reviewed by Holen and co-workers [123].

### Biomarkers of eggs and processed eggs

From the initial systematic literature review process, a total of 635 papers on egg intervention and egg consumption were found. From these, 507 papers remained after combining the results and removing duplicates. After a careful reading and review of the collected literature, only 30 articles were found to be relevant. However, 6 articles were excluded because the dietary egg was not the primary aim of the intervention. On the other hand, 5 additional papers were found in the secondary literature search. From this set of 29 articles, 13 focused on “enriched eggs”, which were not included in the table of potential biomarkers since our focus was on biomarkers of regular eggs. At the end of this selection process, 16 papers were further evaluated in searching for useful biomarkers of egg intake (Additional file 1: Table S9). However, no promising candidates were found for egg consumption; therefore none of the metabolites presented in Additional file 1: Table S9 was tabulated as a candidate BFI in the main body of this review.

#### Eggs and processed eggs

Eggs are widely acknowledged as an excellent source of high-quality protein, providing the richest mix of essential amino acids, vitamins and minerals [124]. Eggs are among the richest sources of dietary cholesterol. However, the relatively high abundance of cholesterol in eggs has limited their consumption. Although dietary recommendations for egg intake are often based on their cholesterol content, the absorption efficiency of dietary cholesterol has been shown to be highly variable between individuals (from 15 to 85%) [125]. In contrast to cholesterol, which is colourless and animal-derived, the carotenoids lutein and zeaxanthin are plant-derived pigments that confer yellow, orange and red colours to the egg yolk. Such compounds are also present in fruits and vegetables, such as corn, kale, leeks, peas, lettuce, carrots, broccoli, basil and parsley [126, 127], and their presence in eggs reflects the presence of plant products in the hens’ diet, as birds are not able to synthesize them. Although the content of these compounds is higher in plants than in eggs, egg-derived carotenoids are significantly more bioavailable than those found in lutein-rich vegetables, such as spinach [128].

#### Whole eggs, egg yolk and egg by-products

Early studies showed that egg consumption was associated with increased levels of low density lipoprotein (LDL) [129, 130]. LDL is the major carrier of dietary cholesterol and cholesterol esters in the human body. LDL (as well as other lipoprotein) measurements therefore provide the best measure of cholesterol intake. Egg yolk contains significant amounts of cholesterol, and Beynen et al. [131] measured increased levels of both LDL and high-density lipoprotein (HDL) in subjects who followed a diet rich in egg yolk (6 egg yolks/day for 10 days). Dietary cholesterol accounts for approximately one third of the pooled body cholesterol, the remaining being synthesized in the body [132]. In line with this fact, modest increases in plasma lipoprotein profiles were observed in two other studies [133, 134]. It is important to note that the majority of these early studies used unrealistically large amounts of eggs. Later studies, using a more realistic number of eggs, found an increase in plasma LDL as well as HDL [135–138]. However, the LDL/HDL ratio, an important risk factor for coronary heart disease, was not changed [135]. Because cholesterol and cholesterol esters are present in many other foods, including shellfish, red meat, fish, milk, cheese and butter [139, 140], plasma cholesterol (i.e. LDL or HDL content) is not a specific and sensitive biomarker for egg.

In addition to cholesterol, lutein and zeaxanthin have also been investigated as BFIs for egg consumption. In several crossover studies, both molecules showed significant increases in plasma [141, 142] and the macular retina [143]. Several independent crossover studies also found increases in serum lutein and zeaxanthin after egg consumption [144–146]. The authors also suggested a relationship between lipoprotein and lutein levels after finding that egg consumption increased the concentration of HDL and LDL particles containing lutein and zeaxanthin [145, 146]. However, lutein and zeaxanthin cannot be viewed as specific biomarkers of egg intake since they are also present in many vegetable sources, such as corn, carrots, peppers and green leafy vegetables.

#### Enriched eggs

Eggs are increasingly being enriched to contain higher levels of key health-promoting nutrients such as omega 3 (ω-3) fatty acids, including alpha-linolenic acid (ALA), docosahexaenoic acid (DHA) and eicosapentaenoic acid (EPA). Enrichment has also been extended to lutein, lycopene, vitamin E and selenium. Egg enrichment is obtained by modifying the hen’s diet to incorporate the target nutrient or precursors thereof. In many cases, this is done by feeding hens flaxseeds [147].

Consumption of lutein-enriched eggs has shown increased levels of lutein in human plasma [128, 148, 149]. A number of studies have employed DHA-enriched eggs and tracked the prevalence of DHA in plasma [150]. On the other hand, two studies utilizing ALA-enriched eggs found no correlation between ALA levels in the blood and the levels of egg consumption. Instead, a marked increase in DHA was observed [147] along with increased total triglycerides [151].

One of the first studies to use ω-3-enriched eggs showed that ω-3 fatty acids were significantly enriched in breast milk upon consumption of two ω-3-enriched eggs for 6 weeks [152]. Furthermore, this intervention did not alter plasma cholesterol nor plasma triglyceride levels. A similar effect was seen in two other studies in which EPA [153] and DHA [153, 154] were significantly increased. Conversely, other studies on ω-3 enriched eggs found a reduction in triglyceride levels [155]. Interestingly, a more recent study did not observe any increase in ω-3 fatty acids [156].

In conclusion, egg consumption and their effects on plasma composition have been studied for many years. Early studies of egg intake largely evaluated dietary cholesterol. Although eggs are a rich source of cholesterol, other animal sources contain cholesterol as well. Thus, plasma cholesterol and plasma LDL/HDL levels are not useful biomarkers of egg intake. Most recent studies have switched from evaluating plasma cholesterol content to evaluating the presence of molecules such as carotenoids. However, carotenoids cannot be considered as specific biomarkers of egg intake since they are also present in many vegetable sources. While enriched eggs contain relatively high amounts of low abundance micronutrients, the use of these molecules as BFIs should not be considered as general biomarkers for the intake of eggs or enriched eggs. To date, a sensitive and specific biomarker of egg consumption has not yet been reported in the literature.

## Conclusions

Gao et al. [2] describe three major classes of dietary and health biomarkers: (i) exposure and intake biomarkers reflecting the level of extrinsic variables that humans are exposed to; (ii) effect biomarkers referring to the functional response of the human body to an exposure; (iii) susceptibility biomarkers representing the individual resilience to an exposure. BFIs belong to the first class of biomarkers and their applications depend on their kinetic behaviour. Short term markers (e.g. galactonate for dairy products) reflect recent intake and can be used to assess the validity of dietary assessment methods such as 24 h recall or food diaries and multiple assessments can be used to assess habitual diet. On the other hand, long-term intake (e.g. C15:0 for dairy products) reflect habitual intake. Gao et al. [2] propose a flexible definition of biomarkers, their classification depending on the intended use. In that regard, some of the candidate BFIs described in this review might also be interesting susceptibility or effect biomarker useful to link disease risk to habitual diet.

However, although many compounds have been associated with the intake of dairy or egg products, no reliable biomarkers have been identified so far and the candidate markers reported in this review (Table 1) need further investigation since validation criteria were not fully fulfilled by any of them (Table 2). Table 2 highlights the aspects which still need to be clarified or investigated to fully validate the proposed biomarkers. Lipids such as C17:0, C:15:0, C17:1 and myristoyl-sphingomyelin as well as a galactose metabolite (galactonate) are the most promising candidates for dairy products in general, methyl palmitate for butter and amino acid derivatives for cheese (Table 1). Instead, for milk, milk proteins and fermented dairy products, including yogurt, the evidence for potentially interesting BFIs is scarce.

The identification of BFIs for dairy products is quite challenging, as this food group is characterized by marketed products with a highly heterogeneous composition resulting from differences in processing (e.g. fermentation, starter cultures, storage conditions and fat content), as well as in the quality of the milk used as raw material (season, feeding process, stage of lactation, region). Moreover, the candidate markers may originate from external sources, including the plants on which the animals are fed, so that these markers may also be found in meat and meat products. Therefore, the reliable marker should be sufficiently robust and specific to discriminate among different foods of animal origin. The candidate markers summarized in this review derive from particular compounds present in foods of animal origin (e.g. ruminant fatty acids) or resulting from food processing (e.g. cyclic dipeptides for hydrolyzed whey protein). The validation scheme shown in Table 2 clearly shows that none of these markers are specific enough for the individual food investigated (question 1). However, a combination of markers, which reflect different characteristics of the food of interest (e.g. fat content, food processing, animal feeding, etc.) could provide a better estimation of dairy intake. Moreover, the results of the literature search pointed out that the number of studies investigating a specific class of dairy products (e.g. cheese, milk or yogurt) is quite limited (Additional file 1: Tables S3–S8) and information regarding quantitative aspects and kinetic is lacking (questions 2 and 3, respectively). Intervention studies based on untargeted approaches should also be designed in order to evaluate the sensitivity and specificity of markers, which are able to discriminate among different dairy products, particularly among different fermented products (e.g. cheese and yogurt or different varieties of cheese), between butter and other high-fat dairy products, as well as between dairies and other ruminant foods. Moreover, the heterogeneity in these foods may invalidate their use as universal markers for different situations, e.g. for populations in different geographical areas. Therefore, further studies should test the candidate biomarkers for dairy intake in different populations (question 4).

As for dairy products, sensitive and specific biomarkers for egg consumption have not yet been reported. The studies evaluated in this review focused on compounds (cholesterol and carotenoids) targeted because of their functional properties, rather than their potential as BFIs. Also, untargeted metabolomics approaches have not been carried out so far. Moreover, the targeted studies revealed that the nutritional content of eggs is highly influenced by the animal’s diet, what represents a source of variation that needs to be taken into account in further investigations. Acute untargeted and pharmacokinetic studies are therefore needed to identify suitable, specific and sensitive biomarkers of egg and egg-derived product intake.

Finally, both for dairy products and egg products, blood (plasma or serum) was the main biological sample investigated; new studies should therefore address other matrices, in particular, urine.

## Additional files

Additional file 1: **Tables S1-S10.** Describing the literature search criteria and the lists of studies and putative biomarkers of intake of dairy and egg products. (DOCX 138 kb)

Additional file 2: **Figure S1.** Overview of primary literature search. (PPTX 79 kb)
