# Supplementary material for: Biomarker of food intake for assessing the consumption of dairy and egg products
Source: Genes Nutr. 2018 Sep 29;13:26. doi: 10.1186/s12263-018-0615-5 (PMC6162878; doi:10.1186/s12263-018-0615-5)
Supplement: Supplementary file 1 — Tables S1-S10. Describing the literature search criteria and the lists of studies and putative biomarkers of intake of dairy and egg products. (DOCX 138 kb) [file 12263_2018_615_MOESM1_ESM.docx]

**Additional file 1.** Tables S1-S10 describing the literature search criteria and the lists of studies and putative biomarkers of intake of dairy and egg products

**Table S1.** Common keywords

| **Operator** | **Database** | **Field** | **Keywords** |
| --- | --- | --- | --- |
|  | *Pubmed* | *All Fields* | biomarker* OR marker* OR metabolite* OR biokinetics OR biotransformation |
|  | *Web of Science* | *Topic* |  |
|  | *Scopus* | *Article Title/ Abstract/ Keywords* |  |
| AND | *Pubmed* | *All Fields* | trial OR experiment OR study OR intervention |
|  | *Web of Science* | *Topic* |  |
|  | *Scopus* | *Article Title/ Abstract/ Keywords* |  |
| AND | *Pubmed* | *All Fields* | human* OR men OR women OR patient* OR volunteer* OR participant* |
|  | *Web of Science* | *Topic* |  |
|  | *Scopus* | *Article Title/ Abstract/ Keywords* |  |
| AND | *Pubmed* | *All Fields* | urine OR plasma OR serum OR blood OR excretion |
|  | *Web of Science* | *Topic* |  |
|  | *Scopus* | *Article Title/ Abstract/ Keywords* |  |
| AND | *Pubmed* | *All Fields* | intake OR meal OR diet OR ingestion OR consumption OR eating OR drink* OR administration |
|  | ***Web of Science*** | *Topic* |  |
|  | ***Scopus*** | *Article Title/ Abstract/ Keywords* |  |

**Table S2.** Specific keywords for each food group

| **Dairy products** | | | |
| --- | --- | --- | --- |
| **Dairy products in general*** | | | |
| **Operator** | **Database** | **Field** | **Keywords** |
| AND | *Pubmed* | *All Fields* | Dairy |
|  | *Web of Science* | *Topic* |  |
|  | *Scopus* | *Article Title/ Abstract/ Keywords* |  |
| NOT | *Pubmed* | *All Fields* | "breast milk" OR "breast feeding" OR bone OR muscle OR allerg* OR "phyto ster*" OR "plant ster*" OR phytoster* OR newborn* OR infant* |
|  | *Web of Science* | *Topic* |  |
|  | *Scopus* | *Article Title/ Abstract/ Keywords* |  |
| Milk | | | |
| Operator | Database | Field | Keywords |
| AND | *Pubmed* | *Abstract/title** | Milk |
|  | *Web of Science* | *Topic* |  |
|  | *Scopus* | *Article Title/ Abstract/ Keywords* |  |
| NOT | *Pubmed* | *All Fields* | "breast milk" OR "breast feeding" OR bone OR muscle OR allerg* OR "phyto ster*" OR "plant ster*" OR phytoster* OR newborn* OR infant* |
|  | *Web of Science* | *Topic* |  |
|  | *Scopus* | *Article Title/ Abstract/ Keywords* |  |
| **Butter** | | | |
| **Operator** | **Database** | **Field** | **Keywords** |
| AND | *Pubmed* | *All Fields* | Butter |
|  | *Web of Science* | *Topic* |  |
|  | *Scopus* | *Article Title/ Abstract/ Keywords* |  |
| NOT | *Pubmed* | *All Fields* | "breast milk" OR "breast feeding" OR bone OR muscle OR allerg* OR "phyto ster*" OR "plant ster*" OR phytoster* OR newborn* OR infant* |
|  | *Web of Science* | *Topic* |  |
|  | *Scopus* | *Article Title/ Abstract/ Keywords* |  |
| **Cheese** | | | |
| **Operator** | **Database** | **Field** | **Keywords** |
| AND | *Pubmed* | *All Fields* | Cheese |
|  | *Web of Science* | *Topic* |  |
|  | *Scopus* | *Article Title/ Abstract/ Keywords* |  |
| NOT | *Pubmed* | *All Fields* | "breast milk" OR "breast feeding" OR bone OR muscle OR allerg* OR "phyto ster*" OR "plant ster*" OR phytoster* OR newborn* OR infant* |
|  | *Web of Science* | *Topic* |  |
|  | *Scopus* | *Article Title/ Abstract/ Keywords* |  |

| **Yogurt** | | | |
| --- | --- | --- | --- |
| **Operator** | **Database** | **Field** | **Keywords** |
| AND | *Pubmed* | *All Fields* | yogurt OR yoghurt OR yoghourt |
|  | *Web of Science* | *Topic* |  |
|  | *Scopus* | *Article Title/ Abstract/ Keywords* |  |
| **Fermented non-solids dairy products** | | | |
| **Operator** | **Database** | **Field** | **Keywords** |
| AND | *Pubmed* | *All Fields* | quark OR “sour cream” OR kefir OR buttermilk |
|  | *Web of Science* | *Topic* |  |
|  | *Scopus* | *Article Title/ Abstract/ Keywords* |  |
| NOT | *Pubmed* | *All Fields* | "breast milk" OR "breast feeding" OR bone OR muscle OR allerg* OR "phyto ster*" OR "plant ster*" OR phytoster* OR newborn* OR infant* |
|  | *Web of Science* | *Topic* |  |
|  | *Scopus* | *Article Title/ Abstract/ Keywords* |  |
| **Whey** | | | |
| **Operator** | **Database** | **Field** | **Keywords** |
| AND | *Pubmed* | *All Fields* | Whey |
|  | *Web of Science* | *Topic* |  |
|  | *Scopus* | *Article Title/ Abstract/ Keywords* |  |
| NOT | *Pubmed* | *All Fields* | "breast milk" OR "breast feeding" OR bone OR muscle OR allerg* OR "phyto ster*" OR "plant ster*" OR phytoster* OR newborn* OR infant* |
|  | *Web of Science* | *Topic* |  |
|  | *Scopus* | *Article Title/ Abstract/ Keywords* |  |
| **Casein** | | | |
| **Operator** | **Database** | **Field** | **Keywords** |
| AND | *Pubmed* | *All Fields* | Casein* |
|  | *Web of Science* | *Topic* |  |
|  | *Scopus* | *Article Title/ Abstract/ Keywords* |  |
| NOT | *Pubmed* | *All Fields* | "breast milk" OR "breast feeding" OR bone OR muscle OR allerg* OR "phyto ster*" OR "plant ster*" OR phytoster* OR newborn* OR infant* |
|  | *Web of Science* | *Topic* |  |
|  | *Scopus* | *Article Title/ Abstract/ Keywords* |  |
|  | *Web of Science* | *Topic* |  |
|  | *Scopus* | *Article Title/ Abstract/ Keywords* |  |

| **Eggs** | | | |
| --- | --- | --- | --- |
| **Operator** | **Database** | **Field** | **Keywords** |
| AND | *Pubmed* | *All Fields* | egg OR eggs OR omelet OR omelette |
|  | *Web of Science* | *Topic* |  |
|  | *Scopus* | *Article Title/ Abstract/ Keywords* |  |
| NOT | *Pubmed* | *All Fields* | infect* OR parasitol* OR schistosoma OR egg development OR vaccine OR immune |
|  | *Web of Science* | *Topic* |  |
|  | *Scopus* | *Article Title/ Abstract/ Keywords* |  |
| NOT | *Pubmed* | *All Fields* | pesticide OR pollutant OR dioxins OR contamina* OR arsenic OR perchlorate OR perfluoro* |
|  | *Web of Science* | *Topic* |  |
|  | *Scopus* | *Article Title/ Abstract/ Keywords* |  |
| NOT | *Pubmed* | *All Fields* | oocyte OR fertilization OR hormone |
|  | *Web of Science* | *Topic* |  |
|  | *Scopus* | *Article Title/ Abstract/ Keywords* |  |
| NOT | *Pubmed* | *All Fields* | cytokine OR enzyme OR antibody OR toxicol* OR liposome |
|  | *Web of Science* | *Topic* |  |
|  | *Scopus* | *Article Title/ Abstract/ Keywords* |  |
| NOT | *Pubmed* | *All Fields* | “egg allergy” |
|  | *Web of Science* | *Topic* |  |
|  | *Scopus* | *Article Title/ Abstract/ Keywords* |  |

* The dairy section includes also the results from the search for “milk”, “cheese”, “butter”, “fermented non-solids dairy products” and “yogurt”, where the effect of the single food couldn’t be distinguished from the others.

**Table S3.** List of selected studies and putative biomarkers of intake for dairy products

| Dietary factor | Study design | ^1^N | ^2^Meth | Sample type | Discriminating metabolites / Candidate biomarkers | ^3^Ref |
| --- | --- | --- | --- | --- | --- | --- |
| Dairy fat  (from milk, cream, cheese, ice cream and butter) | Observational study / 7-d dietary record | 62 | GLC | Blood serum | C15:0 in cholesterol ester and phospholipids | [1] |
| Dairy fat  (from milk, sour milk, yogurt, cheese, cream and butter) | Observational study / 1-w dietary record | 114 | GLC | Blood serum (and adipose tissue) | C15:0 in cholesterol esters and phospholipids (C15:0 in adipose tissue)  C17:0 in cholesterol esters and serum phospholipids  14:0 in cholesterol esters and serum phospholipids | [2] |
| Dairy fat  (from milk, cream, ice cream, yoghurt, cheese and butter) | Observational study / 14-d weighed records and FFQ | 110 | GLC | Blood serum (and adipose tissue) | C15:0 in total serum lipids (and adipose tissue) | [3] |
| Dairy fat  (from milk, yoghurt, cream, cheese, ice-cream and butter) | Observational study / 7-d dietary record | 301 | GLC | Blood serum (and adipose tissue) | C15:0 in phospholipids (and adipose tissue)  C17:0 in phospholipids  14:0 in serum phospholipids | [4] |
| Dairy fat  (from milk, sour milk, yogurt, cheese, cream and butter) | Case-control study / FFQ | 111 cases + 107 controls | GC-FID | Blood serum (and adipose tissue) | C15:0 in triacylgylcerols and cholesterol esters (and adipose tissue)  C17:1 in non-esterified fatty acids, triacylgylcerols and cholesteryl esters | [5] |
| Dairy fat  (from milk, cream, sherbet, yogurt, ice cream, cheese, butter and foods with milk ingredients) | Case-control study / FFQ | 166 IHD cases + 327 controls | GC | Blood plasma and erythrocytes | C15:0 in total lipids  *Trans-*16:1n-7 in total lipids | [6] |
| Dairy products | Cross-sectional study / diet history questionnaire | 1114 | GC | Blood serum | C15:0 in phospholipids | [7] |
| Dairy products (milk, yogurt, and cheese) | Cross-sectional study / 24-h dietary recalls and FFQ | 3009 | GC | Blood plasma | C15:0 and C17:0 in phospholipids (only at individual level) | [8] |
| Whole-fat dairy | Prospective cohort study / FFQ | 2837 | GC-FID | Blood plasma | C15:0 in phospholipids | [9] |
| Butter + high fat dairy products | Cross-sectional study / FFQ | 2380 | (FIA)-MS/MS (targeted) | Blood serum | Lysophosphatidylcholine C17:0 | [10] |
| Dairy products (cheese, milk, skim milk, buttermilk, cottage and ricotta cheese, ice cream and yogurt) | Cohort study / FFQ | 659 | GC-FID | Blood serum | C15:0 in total lipids | [11] |
| Full fat dairy products | Observational study / 4-d food records | 86 | GC-MS  (lipidomic) | Blood plasma | Lysophosphatidylcholine (C15:0)  and phospholipid fatty acids (C15:0)  Lysophosphatidylcholine (C17:0)  Lyso-platelet-activating factor (20:0 and 22:1)  Phospholipid fatty acids (*trans-*16:1n-7, 18:1n-7) | [12] |
| Dairy products / dairy fat | Cross-sectional study / 4-d diet record | 279 | GLC | Blood plasma | Dairy products:  C15:0 in phospholipids  14:0 in phospholipids  Dairy fat:  C15:0 in phospholipids  C17:0 in phospholipids | [13] |
| Dairy products (butter, cheese, milk, yoghurt and cream) | Observational study / dietary questionnaire | 4232 | GLC | Blood serum | C15:0 in cholesterol ester  C14:0 in cholesterol ester  C18:0 and C18:1n-9 in cholesterol ester | [14] |
| Dairy products  (milk, cream, yoghurt, cheese, butter) | Observational study / FFQ | 1180 | GC-FID | Dried blood spot | C15:0 | [15] |
| Dairy products | Randomized intervention study  (increased  dairy, reduced dairy or no change,  1 month) | 180 | GC-MS and GC-FID | Blood plasma | C15:0 in phospholipids  C17:0 in phospholipids  C14:0 in phospholipids | [16] |
| Dairy fat | Randomized controlled intervention study  (regular fat dairy replaced by low-fat dairy, 12 weeks) | 114 children | ? | Blood serum | C15:0 | [17] |
| Dairy products (cheddar cheese, butter, extra creamy whole milk) | Randomized, cross-over intervention  (breakfast with dairy or soy, postprandial 4h) | 16 | LC-MS  (lipidomics) | Blood plasma | Phosphatidylcholine C15:0  Phosphatidylcholine C17:0  Phosphatidylcholines C29:0, C31:0, C35:1, C28:0, C30:0, C32:0 and C32:1 | [18] |
| Dairy products | Free-living, multi-centre, cross-over intervention study  (3 servings/d: 1% fat milk, 1.5% fat yogurt and 34% fat cheese, 4 weeks) | 124 | GC-FID for FA analysis | Blood plasma | C15:0 in total lipids  C17:0 in total lipids  C18:3n-6, C22:1n-9 and C22:5n-3 in total lipids | [19] |
| Whole-fat dairy | Prospective cohort study / FFQ | 2617 | GC | Blood plasma | Whole fat dairy:  *Trans-*16:1n-7 in phospholipids  C14:0 in phospholipids  C15:0 in phospholipids  Low fat dairy :  C14:0 in phospholipids  C15:0 in phospholipids | [20] |
| Dairy products (total dairy products and milk) | Population-based, prospective study / FFQ | 2091 | GC-FID | Erythro-cytes | *Trans*-18:1 isomers | [21] |
| Dairy products (milk, milk powder, yogurt, ice cream and other dairy products) | Observational study / FFQ | 3107 | GC | Erythro-cytes | *Trans-*18:1 | [22] |
| Dairy fat (from milk cheese, yoghurt, butter) | Cross-sectional study / FFQ | 96 | GLC-MS | Blood plasma | Phytanic acid | [23] |
| Dairy products | Observational study / 24-h recall | 271 | LC-MS | Blood plasma | Trimethylamine-N-oxide (TMAO) | [24] |

^1^N: number of subjects; ^2^Meth: analytical method; ^3^Ref: primary reference(s)

**Table S4.** List of selected studies and putative biomarkers of intake for milk

| Dietary factor | Study design | ^1^N | ^2^Meth | Sample type | Discriminating metabolites / Candidate biomarkers | ^3^Ref |
| --- | --- | --- | --- | --- | --- | --- |
| Milk  (cow, skimmed) | Intervention study / parallel design with meat diet (7 d) | 12 8-year old boys | ^1^H NMR | Urine (24 h) and blood serum | Serum:  SCFA | [25] |
| Acidified Milk  Probiotic acidified (cow, high-pasteurized, 1.5% fat, with *L. delbrueckii*, *S. thermophilus* and 3 probiotic strains)  Non-probiotic acidified milk (cow, high-pasteurized, 1.5% fat, with gluconic acid lactone) | Intervention study / parallel design (8 w) | 61 IBS patients | ^1^H NMR | Blood serum | L-lactate  3-hydroxybutyrate | [26] |
| Acidified Milk  Probiotic acidified (cow, high-pasteurized, 1.5% fat, with *L. delbrueckii*, *S. thermophilus* and 3 probiotic strains)  Non-probiotic acidified milk (cow, high-pasteurized, 1.5% fat, with gluconic acid lactone) | Intervention study / parallel design (8 w) | 61 IBS patients | GC-MS | Blood serum | Lactate  Glutamine  Proline  Creatine/ creatinine  Aspartic acid | [27] |
| Milk  (cow, semi- skimmed, 1.5% fat) | Randomized crossover intervention study (14 d) | 15 | NMR | Urine and faeces | Urine:  Citrate  Faeces:  Glycerol | [28] |
| Milk  (cow) | Nested case-control study | 502 (255 cases: incident colorectal cancer) | UPLC-MS  GC-MS | Blood serum | Homostachydrine | [29] |
| Fatty milk  (cow) | Observational study/ cohort study / 3-d food records | 135 at 1 year, 133 at 2 years, 92 at 3 years | GC | Blood serum | Pentadecanoic acid C15:0  Palmitic acid 16:0  Conjugated linolenic acid (CLA) | [30] |

^1^N: number of subjects; ^2^Meth: analytical method; ^3^Ref: primary reference(s)

**Table S5.** List of selected studies and putative biomarkers of intake for butter

| Dietary factor | Study design | ^1^N | ^2^Meth | Sample type | Discriminating metabolites / Candidate biomarkers | ^3^Ref |
| --- | --- | --- | --- | --- | --- | --- |
| Butter (salted) | Randomized, crossover intervention study (6 w) | 23 | UPLC-QTOF/MS | Urine  (24 h) | 7 unidentified metabolites | [31] |
| Butter | Cross-sectional study / FFQ | 2380 | (FIA)-MS/MS (targeted) | Blood serum | Saturated acylcarnitines (C9:0, C16:0 and C18:0) | [10] |
| Butter | Nested case-control study  / FFQ | 502 (255 cases: incident colorectal cancer) | UPLC-MS  GC-MS | Blood serum | Methyl palmitate  Pentadecanoic acid C15:0  10-undecenoate (11:1n–1) | [29] |

^1^N: number of subjects; ^2^Meth: analytical method; ^3^Ref: primary reference(s)

**Table S6.** List of selected studies and putative biomarkers of intake for cheese

| Dietary factor | Study design | ^1^N | ^2^Meth | Sample type | Discriminating metabolites / Candidate biomarkers | ^3^Ref |
| --- | --- | --- | --- | --- | --- | --- |
| Cheese (yellow, hard cheese from cow’s milk, ‘Samsø’) | Randomized, crossover intervention study (6 w) | 23 | UPLC-QTOF/MS | Urine (24 h) | Indoxyl sulfate  Xanthurenic acid  Tyramine sulfate  4-Hydroxyphenylacetic acid  Isovalerylglutamic acid  Isovalerylglycine  Tiglylglycine  Isobutyrylglycine | [31] |
| Cheese (semi-hard cheese from cow’s milk, 45% fat/ dry weight, Klovborg) | Randomized crossover intervention study (14 d) | 15 | NMR | Urine and faeces | Urine:  proline betaine  tyrosine  Faeces:  butyrate | [28] |

^1^N: number of subjects; ^2^Meth: analytical method; ^3^Ref: primary reference(s)

**Table S7.** List of selected studies and putative biomarkers of intake for whey and casein

| Dietary factor | Study design | ^1^N | ^2^Meth | Sample type | Discriminating metabolites / Candidate biomarkers | ^3^Ref |
| --- | --- | --- | --- | --- | --- | --- |
| Whey protein | 8-wk double-blinded, placebo-controlled, randomized weight-loss intervention | 27 obese women with metabolic syndrome | GC-MS | Blood plasma | No alteration in plasma branched-chained amino acid | [32] |
| Whey protein isolate  Sodium caseinate | Randomized controlled blinded acute study (240 min) | 15 | AA: TRAQ labeling method  Acylcarnitine; flow injection analysis-MS | Blood plasma  Blood plasma | leucine, tryptophan, lysine, and cysteine  tyrosine, phenyl-alanine, and proline | [33] |
| Whey isolate  Calcium caseinate | Randomized, blinded, crossover meal study / acute study (8 hours) | 11 | LC-QTOF/MS | Blood plasma  Urine  Blood plasma  Urine | Leucine/Isoleucine  γ-glutamyl-leucine, γ-  glutamyl-valine, γ-glutamyl-methionine, and propionylcarnitine  Tryptophan  N-acetyl-tyrosine  Citrulline  Methionine sulfoxide*, N-phenylacetyl-methionine  N-phenylacetyl-Methionine sulfoxide, N-phenylacetyl-methionine, β-asp-Leu | [34] |
| Whey hydrolysate  Whey isolate  Alpha-lactalbumine  Caseinoglyco-macropeptide | Randomized, blinded, cross-over meal study / acute study (8 hours) | 11 | LC-QTOF/MS | Blood plasma  Blood plasma  Blood plasma  Blood plasma | cyclic dipeptides (Pro-Thr, Phe-Val, Ile-Val, Leu-Val, Ala-Ile) and other AA metabolites (β-Asp-Leu, pGlu-Pro, pGlu-Leu, pGlu-Val), N-phenylacetyl-methionine (PAM)* and N-phenylacetyl-methionine sulfoxide (PAMSO)*  indolelactic acid, γ-glutamyl-leucine, phenylalanine, kynurenine, tryptophan  tryptophan, phenylalanine, Indolelactic acid, Kynurenine  Proline-proline  Isoleucine-proline | [35] |
| Whey protein | single-blind, randomized block design (120 min) | 18 | HPLC | Blood plasma | α-amino-butyric  acid, alanine, arginine, asparagine, aspartic acid, citrulline, cysteine, glutamic acid, isoleucine, leucine, lysine, methionine,  ornithine, phenylalanine, serine, threonine, tyrosine and valine | [36] |
| Whey | Randomised, single-blnd acute study (120 min) | 9 | AA: HPLC | Blood plasma | Branched amino acids (valine, isoleucine, leucine) and threonine | [37] |

^1^N: number of subjects; ^2^Meth: analytical method; ^3^Ref: primary reference(s)

**Table S8.** List of selected studies and putative biomarkers of intake for yoghurt

| Dietary factor | Study design | ^1^N | ^2^Meth | Sample type | Discriminating metabolites / Candidate biomarkers | ^3^Ref |
| --- | --- | --- | --- | --- | --- | --- |
| Fresh yogurt | Acute & sustained study, crossover (2 wk) | 24 | GC | Plasma (0-3h after intake at the end of the study) | Propionate | [38] |
| Yogurt | Sustained study, crossover (3 wk) | 12 | Cultural count of bacterial colonies | Faeces (fresh sample) | Bifidobacteria | [39] |
| Yogurt | Sustained study, parallel (3 wk) | 28 | Cultural count of bacterial colonies | Faeces (fresh sample) | Lactobacilli  Bifidobacteria | [40] |
| Yogurt | Sustained study, parallel (4 wk) | 33 | HPLC | Plasma (overnight fasting) | Thiamine  Riboflavin | [41] |
| Yogurt enriched with L. acidophilus | Sustained study, placebo-controlled, parallel (6 wk) | 24 | Microbiological assay with L. leichmannii | Plasma (overnight fasting) | Vitamin B12 | [42] |
| Yogurt | Sustained study, parallel (8 wk) | 61 | 1H NMR | Serum | L-lactate  3-Hydroxybutyrate | [26] |
| Yogurt | Cross-sectional study | 366 | Electrochem-iluminescence | Plasma (overnight fasting) | Vitamin B12 | [43] |

^1^N: number of subjects; ^2^Meth: analytical method; ^3^Ref: primary reference(s)

**Table S9.** List of selected studies and putative biomarkers of intake for egg

| Dietary factor | Study design | ^1^N | ^2^Meth | Sample type | Discriminating metabolites / Candidate biomarkers | ^3^Ref |
| --- | --- | --- | --- | --- | --- | --- |
| Extra-large egg | Randomized double-blind crossover (1 egg/d, 3weeks) | 17 lactovegetarian college students | Enzymatic assay  Immunoassay | Plasma | LDL  ApoB | [44] |
| Egg-yolk | 10 days-intervention and repeated 1 year after (6 egg-yolk/d) | 6 healthy subjects | Enzymatic assay | Serum | LDL  HDL  HDL/LDL | [45] |
| Eggs | 3 weeks intervention study | 81 men | Enzymatic assay | Plasma | LDL | [46] |
| Eggs | Randomized, four-way crossover (8 weeks) | 20 young healthy men | Enzymatic assay  Fluid-phase  radioimmunoassay | Plasma | Total cholesterol  LDL  ApoB | [47] |
| Eggs | Randomized, three-way crossover design (8 weeks) | 13 young healthy women | Enzymatic assay | Plasma | Total cholesterol  LDL  HDL  ApoB | [48] |
| Liquid whole eggs | Randomized cross-over | 51 pre-menopausal women | Enzymatic assay | Plasma | HDL  LDL | [49] |
| Liquid pasteurized whole eggs | Randomized cross-over | 27 premenopausal women and 25 men | Enzymatic assay | Plasma | LDL  CE  TG | [50] |
| Liquid whole eggs | Randomized crossover | 42 elderly subjects | Enzymatic assay | Plasma | HDL  LDL | [51] |
| Whole eggs | 12 week intervention study | 56 young healthy subjects | Enzymatic assay | Plasma | Total cholesterol HDL | [52] |
| Egg-yolk | 4 separate diets in crossover design | 11 moderate hyper-cholesterolemic subjects | HPLC-DAD  Enzymatic assay | Plasma | Lutein  Zeaxanthin  LDL | [53] |
| Liquid whole eggs | Randomized cross-over | 42 elderly subjects | HPLC  Enzymatic assay  NMR | Plasma | Lutein  Zeaxanthin  HDL  LDL | [54] |
| Whole egg | 12 week intervention study | 24 women | HPLC-DAD  Heterochromatic flicker photometry | Serum  Macular retina | Zeaxanthin | [55] |
| Whole egg | Randomized cross-over (18- wk) | 33 men and women | HPLC-DAD | Serum | Lutein  Zeaxanthin | [56] |
| Liquid-pasteurized whole eggs | Randomized crossover (30 days) | 40 men and women hyper- or hypo-responders to dietary cholesterol | HPLC-DAD | Plasma | Lutein  β-carotene | [57] |
| Liquid pasteurized whole eggs | Randomized crossover (30 days) | 22 post-menopausal women | HPLC -DAD | Plasma | Lutein | [58] |
| Liquid whole egg | Randomized, single-blind, placebo-controlled parallel (12 weeks) | 40 men and women with MetS | HPLC-DAD | Plasma  Plasma HDL  Plasma LDL | Lutein  Zeaxanthin | [59] |

^1^N: number of subjects; ^2^Meth: analytical method; ^3^Ref: primary reference(s)

ApoB: Apolipoprotein B, HDL: high density lipoprotein, LDL: low density lipoprotein, VLDL: very low density lipoprotein, IDL: intermediate density lipoprotein, TG: triglycerides, DAD: UV-vis diode array detector; MPOD: macular pigment optical density, MetS: metabolic syndrome, CE: cholesteryl esters.

**Table S10**. Validation scoring system. Based on criteria of Dragsted et al. [60].

| N˚ | Questions |  |  |  |
| --- | --- | --- | --- | --- |
| Biological/nutritional validation and applicability: | |  |  |  |
| 1. | Is the marker compound plausible as a specific BFI for the food or food group (chemical/biological plausibility)? | Y | N | U |
| 2. | Is there a dose-response relationship at relevant intake levels of the targeted food (quantitative aspect)? | Y | N | U |
| 3. | Is the biomarker kinetics described adequately to make a wise choice of sample type, frequency and time window (time-response)? | Y | N | U |
| 4. | Has the marker been shown to be robust after intake of complex meals reflecting dietary habits of the targeted population (robustness)? | Y | N | U |
| 5. | Has the marker been shown to compare well with other markers or questionnaire data for the same food/food group (reliability)? | Y | N | U |
| Analytical validation | |  |  |  |
| 6. | Is the marker chemically and biologically stable during biospecimen collection and storage, making measurements reliable and feasible (stability)? | Y | N | U |
| 7. | Are analytical variability (CV%), accuracy, sensitivity and specificity known as adequate for at least one reported analytical method (analytical performance)? | Y | N | U |
| 8. | Has the analysis been successfully reproduced in another laboratory (reproducibility)? | Y | N | U |

U: uncertain; N: no; Y: yes

**References**

1. Smedman AEM, Gustafsson IB, Berglund LGT, Vessby BOH: Pentadecanoic acid in serum as a marker for intake of milk fat: relations between intake of milk fat and metabolic risk factors. American Journal of Clinical Nutrition 1999, 69:22-29.

2. Wolk A, Furuheim M, Vessby B: Fatty acid composition of adipose tissue and serum lipids are valid biological markers of dairy fat intake in men. Journal of Nutrition 2001, 131:828-833.

3. Brevik A, Veierod MB, Drevon CA, Andersen LF: Evaluation of the odd fatty acids 15 : 0 and 17 : 0 in serum and adipose tissue as markers of intake of milk and dairy fat. European Journal of Clinical Nutrition 2005, 59:1417-1422.

4. Rosell M, Johansson G, Berglund L, Vessby B, de Faire U, Hellenius ML: The relation between alcohol intake and physical activity and the fatty acids 14 : 0, 15 : 0 and 17 : 0 in serum phospholipids and adipose tissue used as markers for dairy fat intake. British Journal of Nutrition 2005, 93:115-121.

5. Biong AS, Berstad P, Pedersen JI: Biomarkers for intake of dairy fat and dairy products. European Journal of Lipid Science and Technology 2006, 108:827-834.

6. Sun Q, Ma J, Campos H, Hu FB: Plasma and erythrocyte biomarkers of dairy fat intake and risk of ischemic heart disease. Am J Clin Nutr 2007, 86:929-937.

7. Thiebaut AC, Rotival M, Gauthier E, Lenoir GM, Boutron-Ruault MC, Joulin V, Clavel-Chapelon F, Chajes V: Correlation between serum phospholipid fatty acids and dietary intakes assessed a few years earlier. Nutr Cancer 2009, 61:500-509.

8. Saadatian-Elahi M, Slimani N, Chajes V, Jenab M, Goudable J, Biessy C, Ferrari P, Byrnes G, Autier P, Peeters PH, et al: Plasma phospholipid fatty acid profiles and their association with food intakes: results from a cross-sectional study within the European Prospective Investigation into Cancer and Nutrition. Am J Clin Nutr 2009, 89:331-346.

9. de Oliveira Otto MC, Nettleton JA, Lemaitre RN, Steffen LM, Kromhout D, Rich SS, Tsai MY, Jacobs DR, Mozaffarian D: Biomarkers of dairy fatty acids and risk of cardiovascular disease in the Multi-ethnic Study of Atherosclerosis. Journal of the American Heart Association 2013, 2.

10. Floegel A, von Ruesten A, Drogan D, Schulze MB, Prehn C, Adamski J, Pischon T, Boeing H: Variation of serum metabolites related to habitual diet: a targeted metabolomic approach in EPIC-Potsdam. Eur J Clin Nutr 2013, 67:1100-1108.

11. Santaren ID, Watkins SM, Liese AD, Wagenknecht LE, Rewers MJ, Haffner SM, Lorenzo C, Hanley AJ: Serum pentadecanoic acid (15:0), a short-term marker of dairy food intake, is inversely associated with incident type 2 diabetes and its underlying disorders. American Journal of Clinical Nutrition 2014, 100:1532-1540.

12. Nestel PJ, Straznicky N, Mellett NA, Wong G, De Souza DP, Tull DL, Barlow CK, Grima MT, Meikle PJ: Specific plasma lipid classes and phospholipid fatty acids indicative of dairy food consumption associate with insulin sensitivity. American Journal of Clinical Nutrition 2014, 99:46-53.

13. Warensjo Lemming E, Nalsen C, Becker W, Ridefelt P, Mattisson I, Lindroos AK: Relative validation of the dietary intake of fatty acids among adults in the Swedish National Dietary Survey using plasma phospholipid fatty acid composition. J Nutr Sci 2015, 4:e25.

14. Laguzzi F, Alsharari Z, Riserus U, Vikstrom M, Sjogren P, Gigante B, Hellenius ML, Cederholm T, Bottai M, de Faire U, Leander K: Cross-sectional relationships between dietary fat intake and serum cholesterol fatty acids in a Swedish cohort of 60-year-old men and women. J Hum Nutr Diet 2016, 29:325-337.

15. Albani V, Celis-Morales C, Marsaux CF, Forster H, O'Donovan CB, Woolhead C, Macready AL, Fallaize R, Navas-Carretero S, San-Cristobal R, et al: Exploring the association of diary product intake with the fatty acids C15:0 and C17:0 measured from dried blood spots in a multi-population cohort: findings from the Food4Me study. Mol Nutr Food Res 2015.

16. Benatar JR, Stewart RA: The effects of changing dairy intake on trans and saturated fatty acid levels- results from a randomized controlled study. Nutr J 2014, 13:32.

17. Golley RK, Hendrie GA: Evaluation of the relative concentration of serum fatty acids C14:0, C15:0 and C17:0 as markers of children's dairy fat intake. Ann Nutr Metab 2014, 65:310-316.

18. Meikle PJ, Barlow CK, Mellett NA, Mundra PA, Bonham MP, Larsen A, Cameron-Smith D, Sinclair A, Nestel PJ, Wong G: Postprandial Plasma Phospholipids in Men Are Influenced by the Source of Dietary Fat. J Nutr 2015, 145:2012-2018.

19. Abdullah MMH, Cyr A, Lepine MC, Labonte ME, Couture P, Jones PJH, Lamarche B: Recommended dairy product intake modulates circulating fatty acid profile in healthy adults: a multi-centre cross-over study. British Journal of Nutrition 2015, 113:435-444.

20. Mozaffarian D, de Oliveira Otto MC, Lemaitre RN, Fretts AM, Hotamisligil G, Tsai MY, Siscovick DS, Nettleton JA: trans-Palmitoleic acid, other dairy fat biomarkers, and incident diabetes: the Multi-Ethnic Study of Atherosclerosis (MESA). Am J Clin Nutr 2013, 97:854-861.

21. Zong G, Sun Q, Yu DX, Zhu JW, Sun L, Ye XW, Li HX, Jin QL, Zheng H, Hu FB, Lin X: Dairy Consumption, Type 2 Diabetes, and Changes in Cardiometabolic Traits: A Prospective Cohort Study of Middle-Aged and Older Chinese in Beijing and Shanghai. Diabetes Care 2014, 37:56-63.

22. Yu DX, Sun Q, Ye XW, Pan A, Zong G, Zhou YH, Li HX, Hu FB, Lin X: Erythrocyte trans-fatty acids, type 2 diabetes and cardiovascular risk factors in middle-aged and older Chinese individuals. Diabetologia 2012, 55:2954-2962.

23. Allen NE, Grace PB, Ginn A, Travis RC, Roddam AW, Appleby PN, Key T: Phytanic acid: measurement of plasma concentrations by gas-liquid chromatography-mass spectrometry analysis and associations with diet and other plasma fatty acids. British Journal of Nutrition 2008, 99:653-659.

24. Rohrmann S, Linseisen J, Allenspach M, von Eckardstein A, Muller D: Plasma Concentrations of Trimethylamine-N-oxide Are Directly Associated with Dairy Food Consumption and Low-Grade Inflammation in a German Adult Population. J Nutr 2016, 146:283-289.

25. Bertram HC, Hoppe C, Petersen BO, Duus JO, Molgaard C, Michaelsen KF: An NMR-based metabonomic investigation on effects of milk and meat protein diets given to 8-year-old boys. British Journal of Nutrition 2007, 97:758-763.

26. Pedersen SMM, Nielsen NC, Andersen HJ, Olsson J, Simren M, Ohman L, Svensson U, Malmendal A, Bertram HC: The Serum Metabolite Response to Diet Intervention with Probiotic Acidified Milk in Irritable Bowel Syndrome Patients Is Indistinguishable from that of Non-Probiotic Acidified Milk by H-1 NMR-Based Metabonomic Analysis. Nutrients 2010, 2:1141-1155.

27. Pedersen SMM, Nebel C, Nielsen NC, Andersen HJ, Olsson J, Simren M, Ohman L, Svensson U, Bertram HC, Malmendal A: A GC-MS-based metabonomic investigation of blood serum from irritable bowel syndrome patients undergoing intervention with acidified milk products. European Food Research and Technology 2011, 233:1013-1021.

28. Zheng H, Yde CC, Clausen MR, Kristensen M, Lorenzen J, Astrup A, Bertram HC: Metabolomics investigation to shed light on cheese as a possible piece in the French paradox puzzle. Journal of Agricultural and Food Chemistry 2015, 63:2830-2839.

29. Guertin KA, Moore SC, Sampson JN, Huang WY, Xiao Q, Stolzenberg-Solomon RZ, Sinha R, Cross AJ: Metabolomics in nutritional epidemiology: identifying metabolites associated with diet and quantifying their potential to uncover diet-disease relations in populations. American Journal of Clinical Nutrition 2014, 100:208-217.

30. Uusitalo L, Nevalainen J, Salminen I, Ovaskainen ML, Kronberg-Kippila C, Ahonen S, Niinisto S, Alfthan G, Simell O, Ilonen J, et al: Fatty acids in serum and diet--a canonical correlation analysis among toddlers. Matern Child Nutr 2013, 9:381-395.

31. Hjerpsted JB, Ritz C, Schou SS, Tholstrup T, Dragsted LO: Effect of cheese and butter intake on metabolites in urine using an untargeted metabolomics approach. Metabolomics 2014, 10:1176-1185.

32. Piccolo BD, Comerford KB, Karakas SE, Knotts TA, Fiehn O, Adams SH: Whey Protein Supplementation Does Not Alter Plasma Branched-Chained Amino Acid Profiles but Results in Unique Metabolomics Patterns in Obese Women Enrolled in an 8-Week Weight Loss Trial. J Nutr 2015, 145:691-700.

33. Hoefle AS, Bangert AM, Stamfort A, Gedrich K, Rist MJ, Lee Y-M, Skurk T, Daniel H: Metabolic Responses of Healthy or Prediabetic Adults to Bovine Whey Protein and Sodium Caseinate Do Not Differ. J Nutr 2015, 145:467-475.

34. Stanstrup J, Schou SS, Holmer-Jensen J, Hermansen K, Dragsted LO: Whey Protein Delays Gastric Emptying and Suppresses Plasma Fatty Acids and Their Metabolites Compared to Casein, Gluten, and Fish Protein. Journal of Proteome Research 2014, 13:2396-2408.

35. Stanstrup J, Rasmussen JE, Ritz C, Holmer-Jensen J, Hermansen K, Dragsted LO: Intakes of whey protein hydrolysate and whole whey proteins are discriminated by LC–MS metabolomics. Metabolomics 2013, 10:719-736.

36. Chungchunlam SMS, Henare SJ, Ganesh S, Moughan PJ: Dietary whey protein influences plasma satiety-related hormones and plasma amino acids in normal-weight adult women. Eur J Clin Nutr 2015, 69:179-186.

37. Hall WL, Millward DJ, Long SJ, Morgan LM: Casein and whey exert different effects on plasma amino acid profiles, gastrointestinal hormone secretion and appetite. British Journal of Nutrition 2003, 89:239-248.

38. Rizkalla SW, Luo J, Kabir M, Chevalier A, Pacher N, Slama G: Chronic consumption of fresh but not heated yogurt improves breath-hydrogen status and short-chain fatty acid profiles: a controlled study in healthy men with or without lactose maldigestion. The American Journal of Clinical Nutrition 2000, 72:1474-1479.

39. Bartram HP, Scheppach W, Gerlach S, Ruckdeschel G, Kelber E, Kasper H: Does yogurt enriched with Bifidobacterium longum affect colonic microbiology and fecal metabolites in health subjects? The American Journal of Clinical Nutrition 1994, 59:428-432.

40. Hussein L, Gouda M, Fouad M, Labib E, Bassyouni R, Mohammad M: Dietary Intervention with Yoghurt, Synbiotic Yogurt or Traditional Fermented Sobya: Bio-Potency among Male Adolescents Using Five Bio-Markers of Relevance to Colonic Metabolic Activities. Food and Nutrition Sciences 2014, 5:1131-1144.

41. Fabian E, Majchrzak D, Dieminger B, Meyer E, Elmadfa I: Influence of Probiotic and Conventional Yoghurt on the Status of Vitamins B1, B2 and B6 in Young Healthy Women. Annals of Nutrition and Metabolism 2008, 52:29-36.

42. Mohammad MA, Molloy A, Scott J, Hussein L: Plasma cobalamin and folate and their metabolic markers methylmalonic acid and total homocysteine among Egyptian children before and after nutritional supplementation with the probiotic bacteria Lactobacillus acidophilus in yoghurt matrix. International Journal of Food Sciences and Nutrition 2006, 57:470-480.

43. Samuel TM, Duggan C, Thomas T, Bosch R, Rajendran R, Virtanen SM, Srinivasan K, Kurpad AV: Vitamin B<sub>12</sub> Intake and Status in Early Pregnancy among Urban South Indian Women. Annals of Nutrition and Metabolism 2013, 62:113-122.

44. Sacks FM, Salazar J, Miller L, Foster JM, Sutherland M, Samonds KW, Albers JJ, Kass EH: Ingestion of egg raises plasma low density lipoproteins in free-living subjects. Lancet 1984, 1:647-649.

45. Beynen AC, Katan MB: Effect of egg yolk feeding on the concentration and composition of serum lipoproteins in man. Atherosclerosis 1985, 54:157-166.

46. Brown SA, Morrisett J, Patsch JR, Reeves R, Gotto AM, Jr., Patsch W: Influence of short term dietary cholesterol and fat on human plasma Lp[a] and LDL levels. J Lipid Res 1991, 32:1281-1289.

47. Ginsberg HN, Karmally W, Siddiqui M, Holleran S, Tall AR, Rumsey SC, Deckelbaum RJ, Blaner WS, Ramakrishnan R: A dose-response study of the effects of dietary cholesterol on fasting and postprandial lipid and lipoprotein metabolism in healthy young men. Arterioscler Thromb 1994, 14:576-586.

48. Ginsberg HN, Karmally W, Siddiqui M, Holleran S, Tall AR, Blaner WS, Ramakrishnan R: Increases in dietary cholesterol are associated with modest increases in both LDL and HDL cholesterol in healthy young women. Arterioscler Thromb Vasc Biol 1995, 15:169-178.

49. Herron KL, Vega-Lopez S, Conde K, Ramjiganesh T, Roy S, Shachter NS, Fernandez ML: Pre-menopausal women, classified as hypo- or hyperresponders, do not alter their LDL/HDL ratio following a high dietary cholesterol challenge. J Am Coll Nutr 2002, 21:250-258.

50. Herron KL, Lofgren IE, Sharman M, Volek JS, Fernandez ML: High intake of cholesterol results in less atherogenic low-density lipoprotein particles in men and women independent of response classification. Metabolism 2004, 53:823-830.

51. Greene CM, Zern TL, Wood RJ, Shrestha S, Aggarwal D, Sharman MJ, Volek JS, Fernandez ML: Maintenance of the LDL cholesterol:HDL cholesterol ratio in an elderly population given a dietary cholesterol challenge. J Nutr 2005, 135:2793-2798.

52. Mayurasakorn K, Srisura W, Sitphahul P, Hongto PO: High-density lipoprotein cholesterol changes after continuous egg consumption in healthy adults. J Med Assoc Thai 2008, 91:400-407.

53. Handelman GJ, Nightingale ZD, Lichtenstein AH, Schaefer EJ, Blumberg JB: Lutein and zeaxanthin concentrations in plasma after dietary supplementation with egg yolk. Am J Clin Nutr 1999, 70:247-251.

54. Greene CM, Waters D, Clark RM, Contois JH, Fernandez ML: Plasma LDL and HDL characteristics and carotenoid content are positively influenced by egg consumption in an elderly population. Nutr Metab (Lond) 2006, 3:6.

55. Wenzel AJ, Gerweck C, Barbato D, Nicolosi RJ, Handelman GJ, Curran-Celentano J: A 12-wk egg intervention increases serum zeaxanthin and macular pigment optical density in women. J Nutr 2006, 136:2568-2573.

56. Goodrow EF, Wilson TA, Houde SC, Vishwanathan R, Scollin PA, Handelman G, Nicolosi RJ: Consumption of one egg per day increases serum lutein and zeaxanthin concentrations in older adults without altering serum lipid and lipoprotein cholesterol concentrations. J Nutr 2006, 136:2519-2524.

57. Clark RM, Herron KL, Waters D, Fernandez ML: Hypo- and hyperresponse to egg cholesterol predicts plasma lutein and beta-carotene concentrations in men and women. J Nutr 2006, 136:601-607.

58. Waters D, Clark RM, Greene CM, Contois JH, Fernandez ML: Change in plasma lutein after egg consumption is positively associated with plasma cholesterol and lipoprotein size but negatively correlated with body size in postmenopausal women. J Nutr 2007, 137:959-963.

59. Blesso CN: Egg Phospholipids and Cardiovascular Health. Nutrients 2015, 7:2731-2747.

60. Dragsted LO, Gao Q, Scalbert A, Vergères G, Kolehmainen M, Manach C, Brennan L, Afman LA, Wishart DS, Andres-Lacueva C, et al: Validation of food intake biomarkers - critical assessment of candidate biomarkers. Genes & Nutrition 2018, 13:14.
