# Supplementary material for: Biomarker of food intake for assessing the consumption of dairy and egg products
Source: Genes Nutr. 2018 Sep 29;13:26. doi: 10.1186/s12263-018-0615-5 (PMC6162878; doi:10.1186/s12263-018-0615-5)
Supplement: Supplementary file 2 — Figure S1. Overview of primary literature search. (PPTX 79 kb) [file 12263_2018_615_MOESM2_ESM.pptx]

## Slide 1
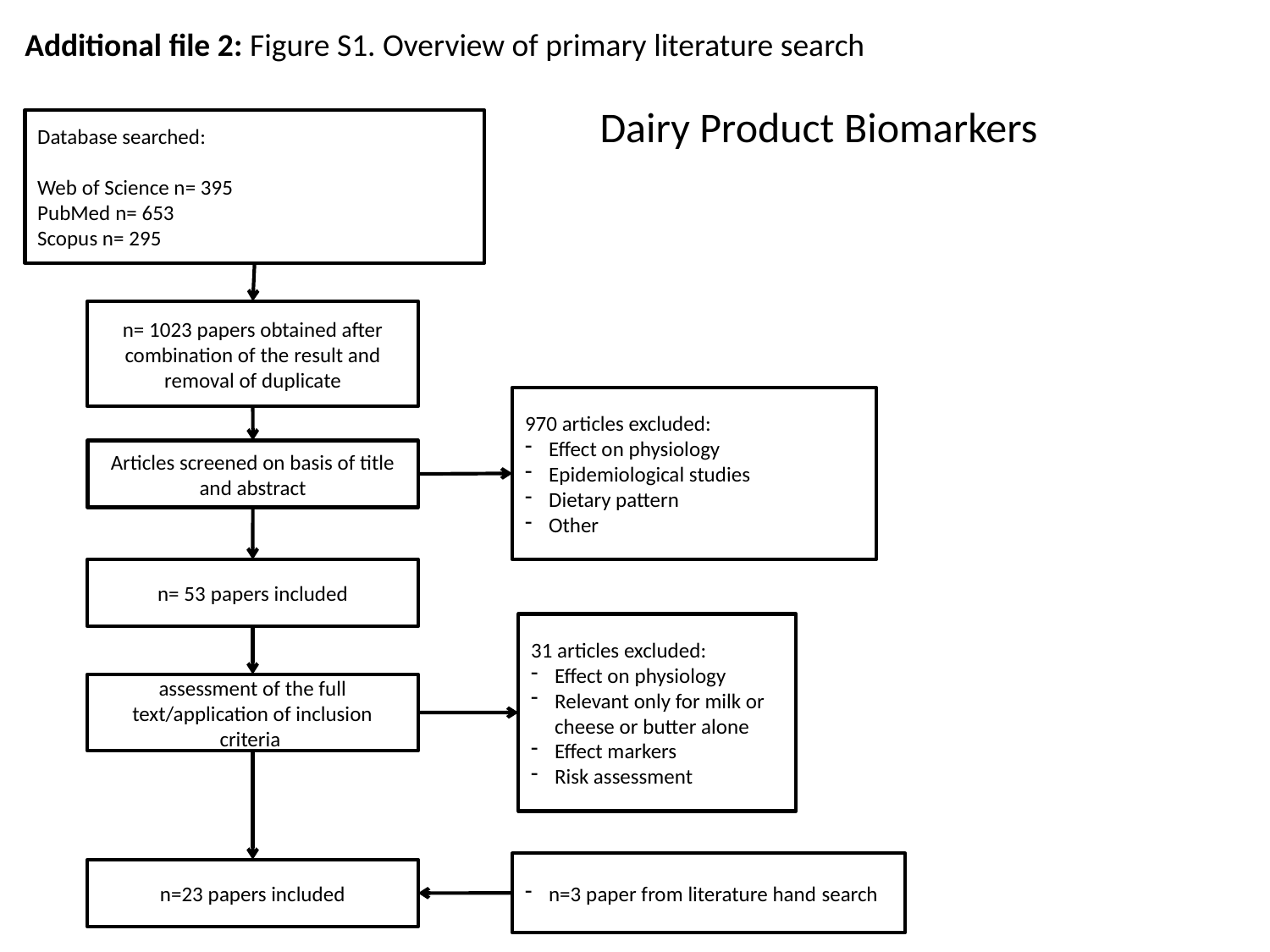

Additional file 2: Figure S1. Overview of primary literature search
Dairy Product Biomarkers
Database searched:
Web of Science n= 395
PubMed n= 653
Scopus n= 295
n= 1023 papers obtained after combination of the result and removal of duplicate
970 articles excluded:
Effect on physiology
Epidemiological studies
Dietary pattern
Other
Articles screened on basis of title and abstract
n= 53 papers included
31 articles excluded:
Effect on physiology
Relevant only for milk or cheese or butter alone
Effect markers
Risk assessment
assessment of the full text/application of inclusion criteria
n=3 paper from literature hand search
n=23 papers included

## Slide 2
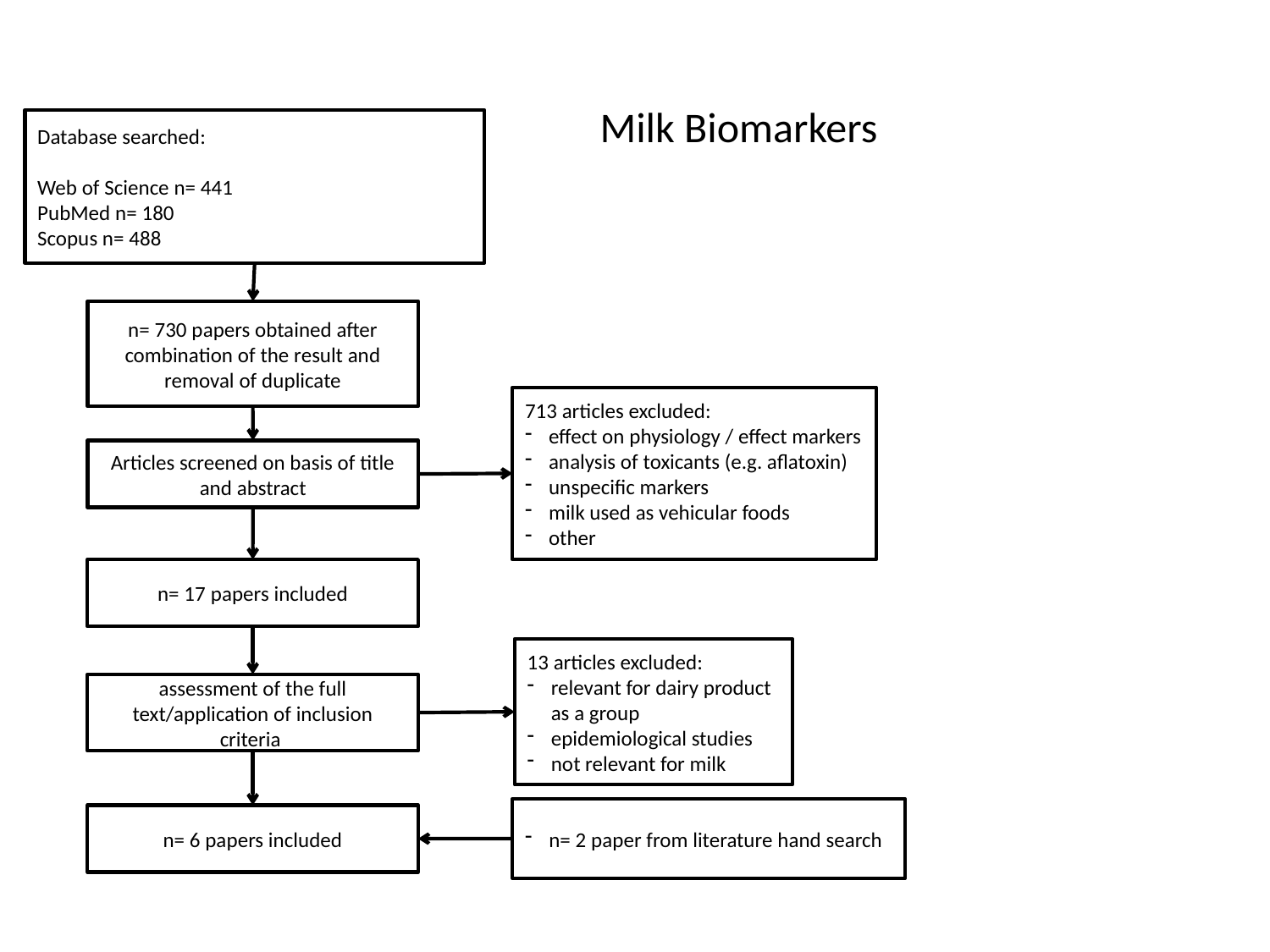

Milk Biomarkers
Database searched:
Web of Science n= 441
PubMed n= 180
Scopus n= 488
n= 730 papers obtained after combination of the result and removal of duplicate
713 articles excluded:
effect on physiology / effect markers
analysis of toxicants (e.g. aflatoxin)
unspecific markers
milk used as vehicular foods
other
Articles screened on basis of title and abstract
n= 17 papers included
13 articles excluded:
relevant for dairy product as a group
epidemiological studies
not relevant for milk
assessment of the full text/application of inclusion criteria
n= 2 paper from literature hand search
n= 6 papers included

## Slide 3
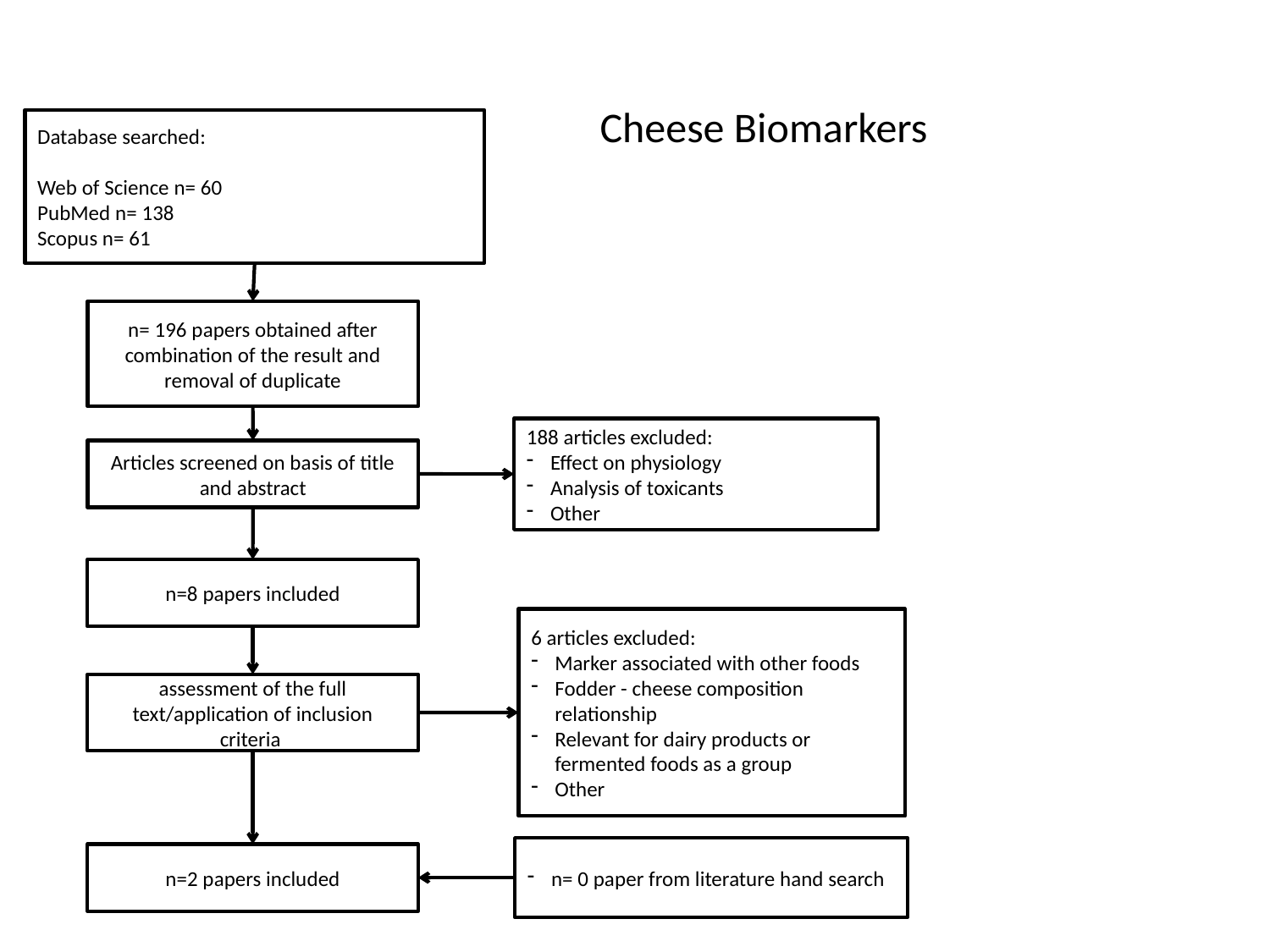

Cheese Biomarkers
Database searched:
Web of Science n= 60
PubMed n= 138
Scopus n= 61
n= 196 papers obtained after combination of the result and removal of duplicate
188 articles excluded:
Effect on physiology
Analysis of toxicants
Other
Articles screened on basis of title and abstract
n=8 papers included
6 articles excluded:
Marker associated with other foods
Fodder - cheese composition relationship
Relevant for dairy products or fermented foods as a group
Other
assessment of the full text/application of inclusion criteria
n= 0 paper from literature hand search
n=2 papers included

## Slide 4
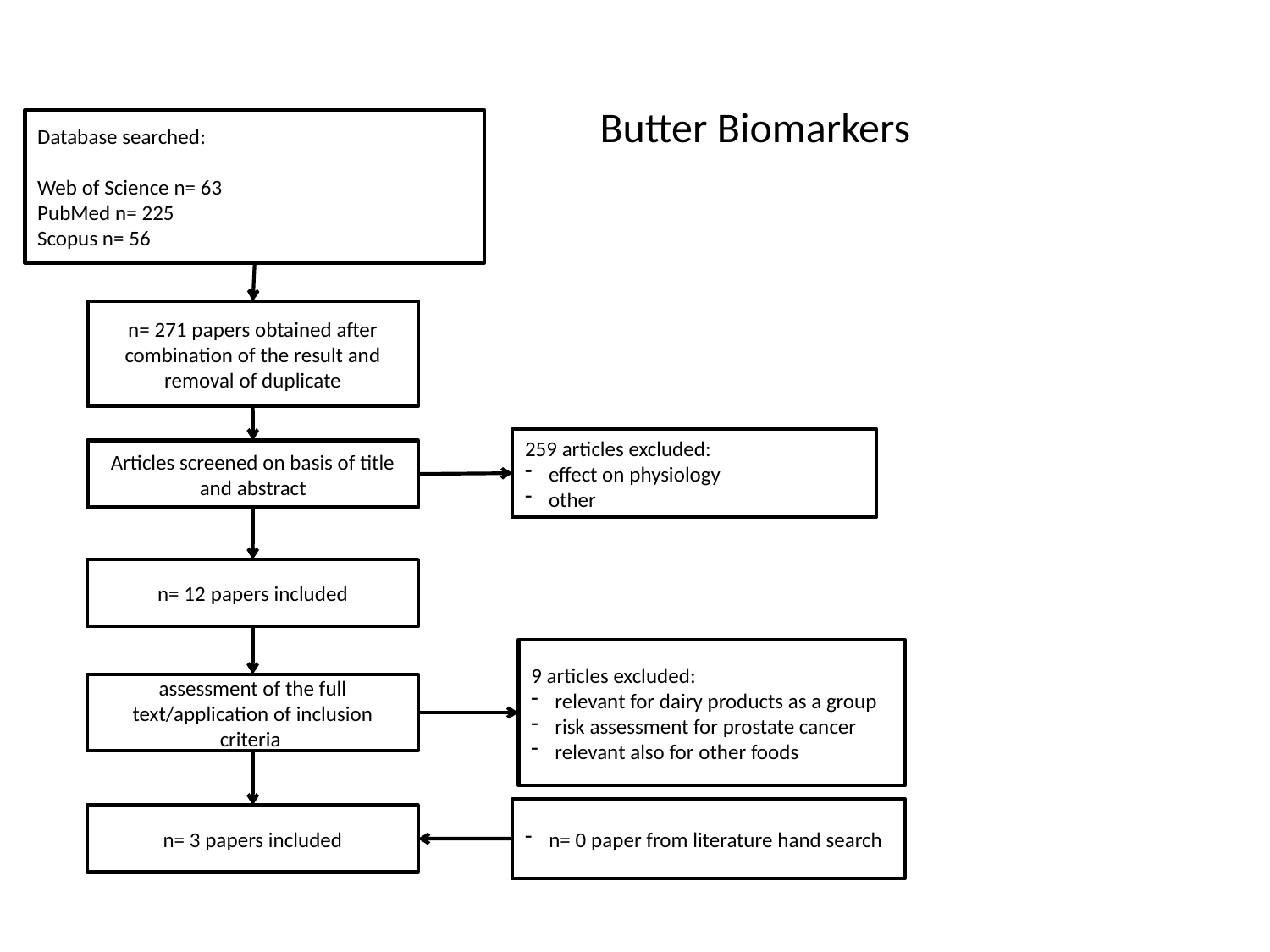

Butter Biomarkers
Database searched:
Web of Science n= 63
PubMed n= 225
Scopus n= 56
n= 271 papers obtained after combination of the result and removal of duplicate
259 articles excluded:
effect on physiology
other
Articles screened on basis of title and abstract
n= 12 papers included
9 articles excluded:
relevant for dairy products as a group
risk assessment for prostate cancer
relevant also for other foods
assessment of the full text/application of inclusion criteria
n= 0 paper from literature hand search
n= 3 papers included

## Slide 5
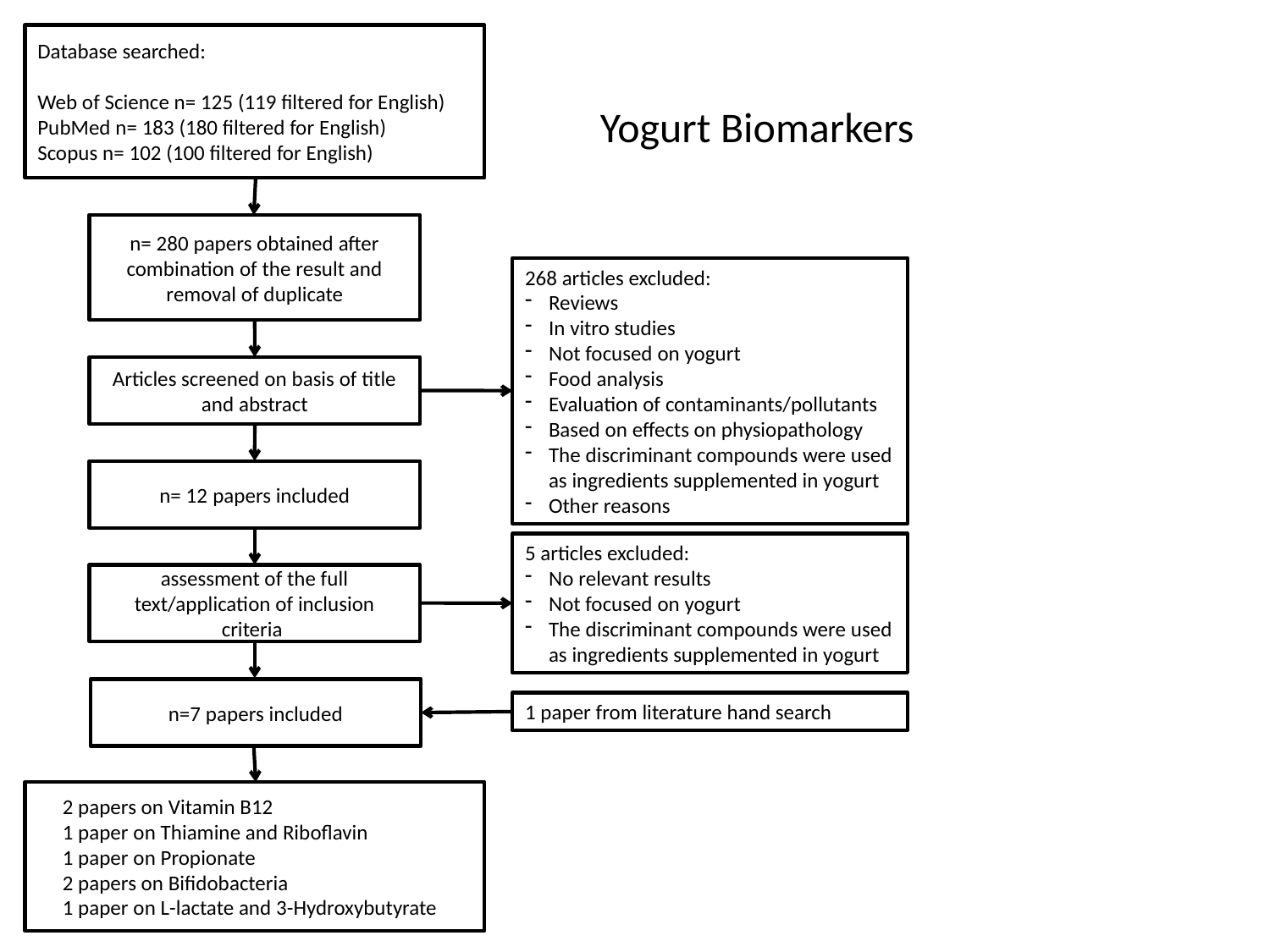

Database searched:
Web of Science n= 125 (119 filtered for English)
PubMed n= 183 (180 filtered for English)
Scopus n= 102 (100 filtered for English)
n= 280 papers obtained after combination of the result and removal of duplicate
268 articles excluded:
Reviews
In vitro studies
Not focused on yogurt
Food analysis
Evaluation of contaminants/pollutants
Based on effects on physiopathology
The discriminant compounds were used as ingredients supplemented in yogurt
Other reasons
Articles screened on basis of title and abstract
n= 12 papers included
5 articles excluded:
No relevant results
Not focused on yogurt
The discriminant compounds were used as ingredients supplemented in yogurt
assessment of the full text/application of inclusion criteria
n=7 papers included
1 paper from literature hand search
2 papers on Vitamin B12
1 paper on Thiamine and Riboflavin
1 paper on Propionate
2 papers on Bifidobacteria
1 paper on L-lactate and 3-Hydroxybutyrate
Yogurt Biomarkers

## Slide 6
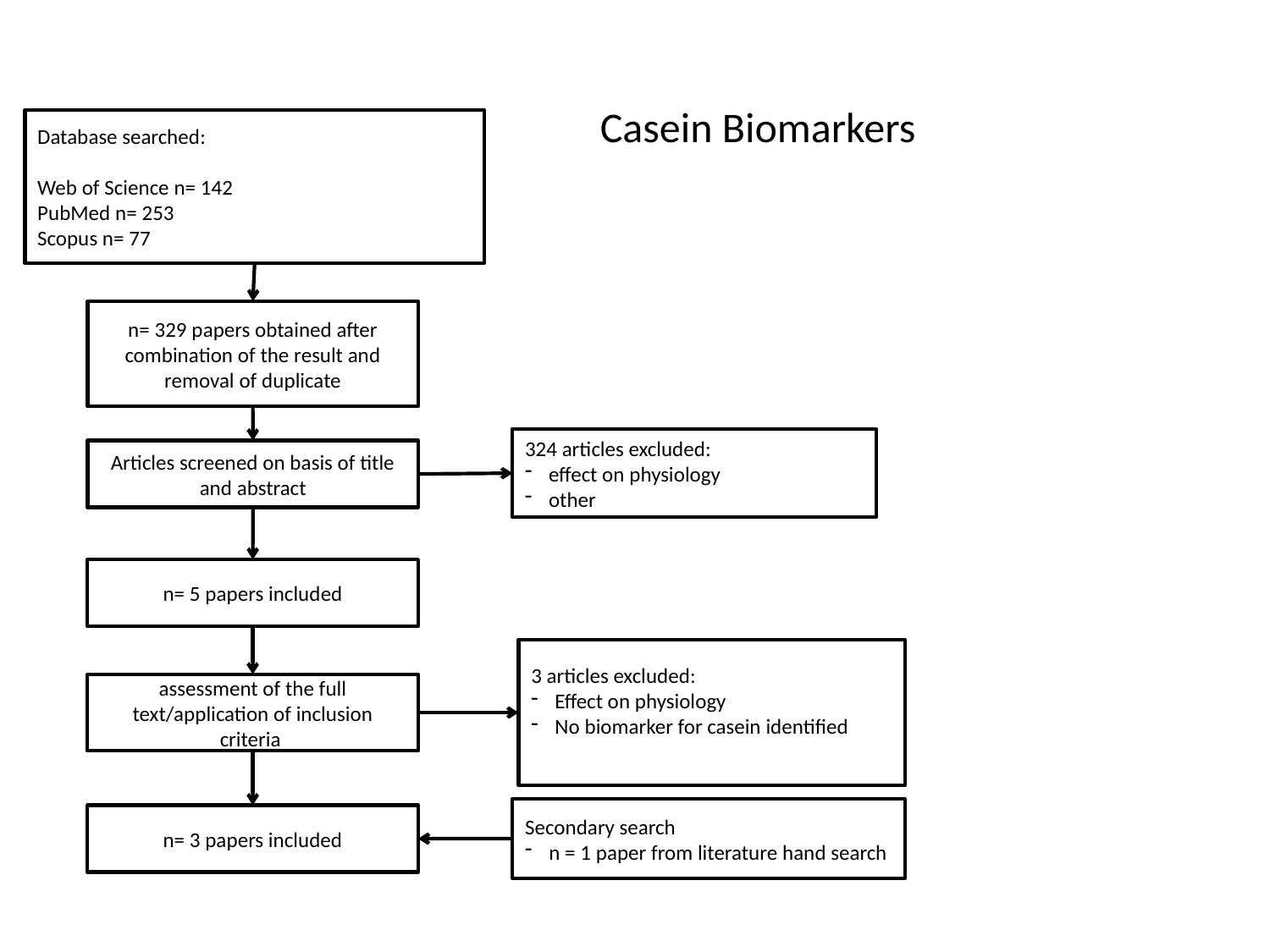

Casein Biomarkers
Database searched:
Web of Science n= 142
PubMed n= 253
Scopus n= 77
n= 329 papers obtained after combination of the result and removal of duplicate
324 articles excluded:
effect on physiology
other
Articles screened on basis of title and abstract
n= 5 papers included
3 articles excluded:
Effect on physiology
No biomarker for casein identified
assessment of the full text/application of inclusion criteria
Secondary search
n = 1 paper from literature hand search
n= 3 papers included

## Slide 7
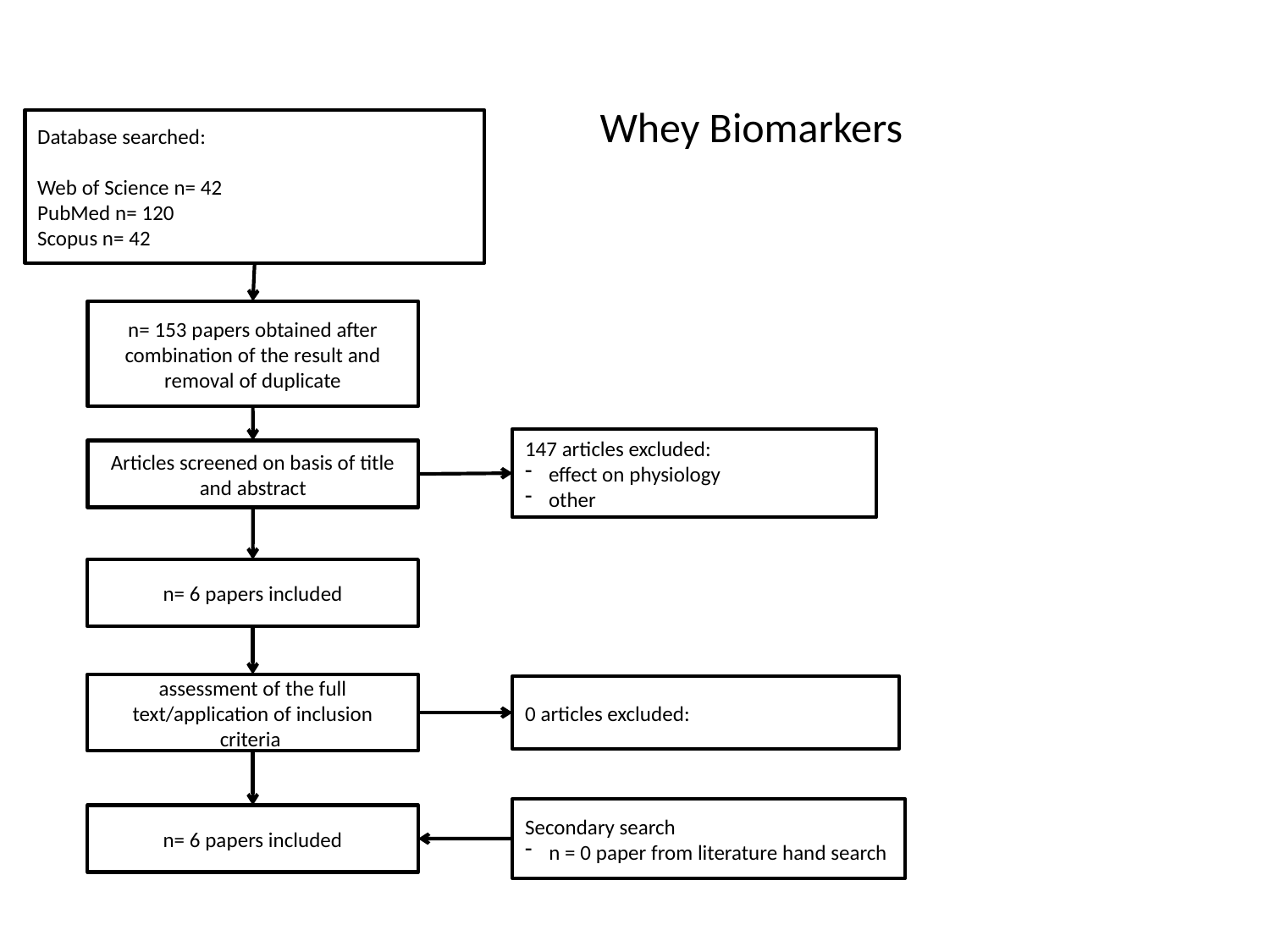

Whey Biomarkers
Database searched:
Web of Science n= 42
PubMed n= 120
Scopus n= 42
n= 153 papers obtained after combination of the result and removal of duplicate
147 articles excluded:
effect on physiology
other
Articles screened on basis of title and abstract
n= 6 papers included
assessment of the full text/application of inclusion criteria
0 articles excluded:
Secondary search
n = 0 paper from literature hand search
n= 6 papers included

## Slide 8
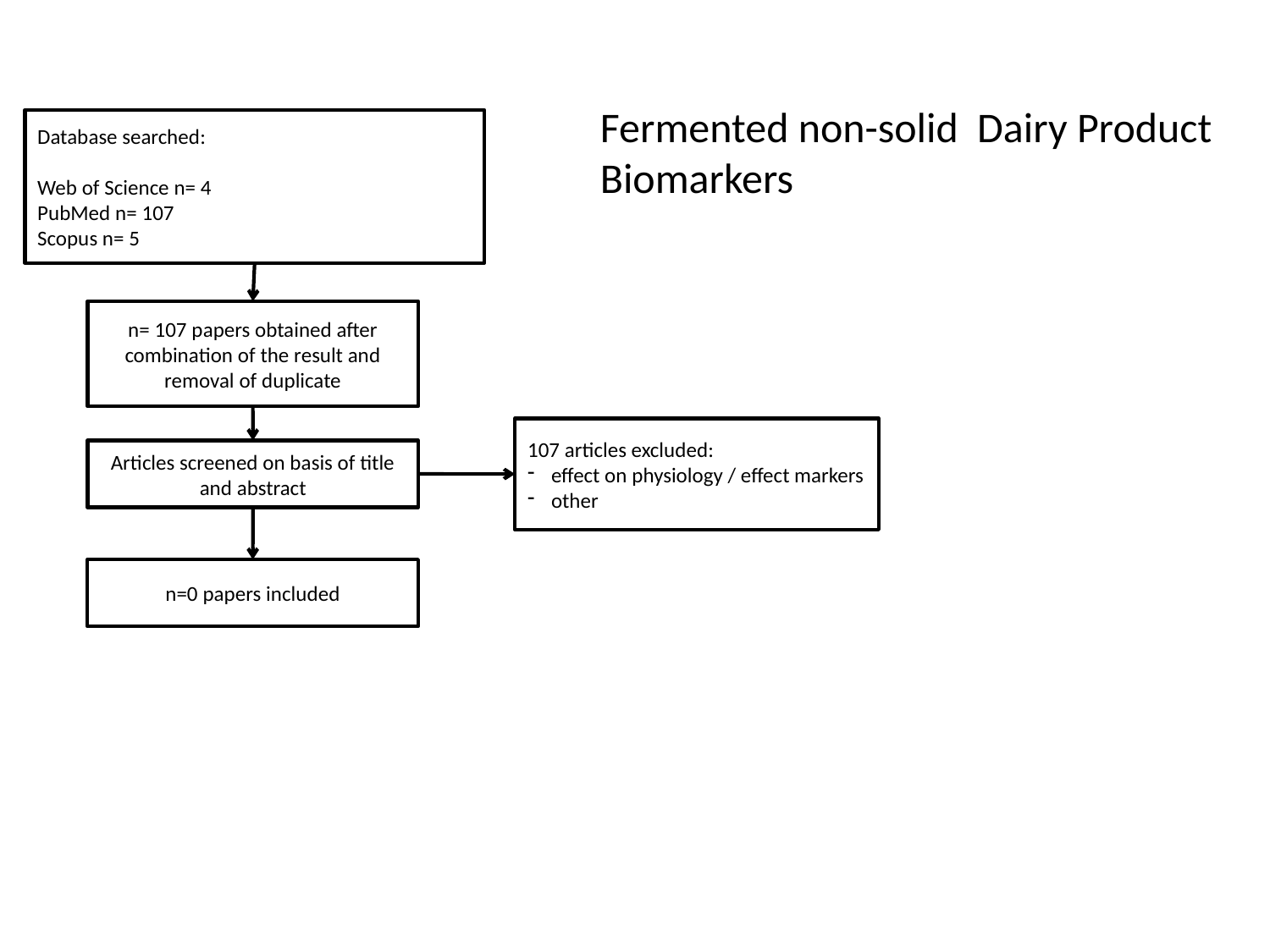

Fermented non-solid Dairy Product Biomarkers
Database searched:
Web of Science n= 4
PubMed n= 107
Scopus n= 5
n= 107 papers obtained after combination of the result and removal of duplicate
107 articles excluded:
effect on physiology / effect markers
other
Articles screened on basis of title and abstract
n=0 papers included

## Slide 9
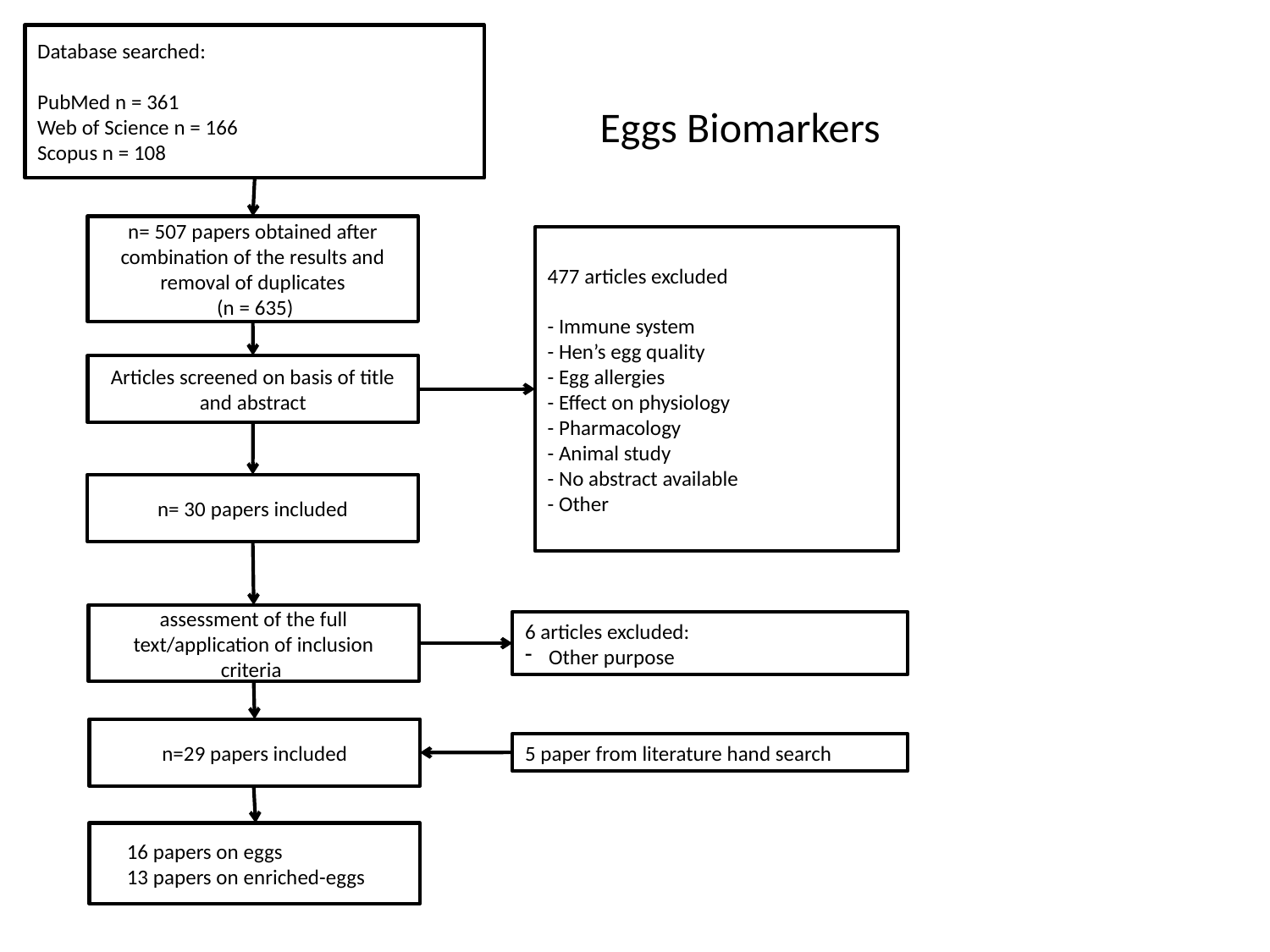

Database searched:
PubMed n = 361
Web of Science n = 166
Scopus n = 108
n= 507 papers obtained after combination of the results and removal of duplicates
 (n = 635)
477 articles excluded
- Immune system
- Hen’s egg quality
- Egg allergies
- Effect on physiology
- Pharmacology
- Animal study
- No abstract available
- Other
Articles screened on basis of title and abstract
n= 30 papers included
assessment of the full text/application of inclusion criteria
6 articles excluded:
Other purpose
n=29 papers included
5 paper from literature hand search
16 papers on eggs
13 papers on enriched-eggs
Eggs Biomarkers
